# Predicting vector distribution in Europe: at what sample size are species distribution models reliable?

**DOI:** 10.3389/fvets.2025.1584864

**Published:** 2025-05-29

**Authors:** Lianne Mitchel, Guy Hendrickx, Ewan T. MacLeod, Cedric Marsboom

**Affiliations:** ^1^Deanery of Biomedical Sciences, College of Medicine and Veterinary Medicine, University of Edinburgh, Edinburgh, United Kingdom; ^2^UK Health Security Agency (UKHSA), Bristol, United Kingdom; ^3^Avia-GIS NV, R&D Department, Zoersel, Belgium; ^4^Spatial Epidemiology Lab (SpELL), Université Libre de Bruxelles (ULB), Brussels, Belgium

**Keywords:** vector-borne diseases, sample size, sample ratio, virtual species, species distribution model, machine learning, random forest, surveillance

## Abstract

**Introduction:**

Species distribution models can predict the spatial distribution of vector-borne diseases by forming associations between known vector distribution and environmental variables. In response to a changing climate and increasing rates of vector-borne diseases in Europe, model predictions for vector distribution can be used to improve surveillance. However, the field lacks standardisation with little consensus as to what sample size produces reliable models.

**Objective:**

Determine the optimum sample size for models developed with the machine learning algorithm, Random Forest, and different sample ratios.

**Materials and methods:**

To overcome limitations with real vector data, a simulated vector with a fully known distribution in 10 test sites across Europe was used to randomly generate different samples sizes. The test sites accounted for varying habitat suitability and the vector’s relative occurrence area. 9,000 Random Forest models were developed with 24 different sample sizes (between 10–5,000) and three sample ratios with varying proportions of presence and absence data (50:50, 20:80, and 40:60, respectively). Model performance was evaluated using five metrics: percentage correctly classified, sensitivity, specificity, Cohen’s Kappa, and Area Under the Curve. The metrics were grouped by sample size and ratio. The optimum sample size was determined when the 25th percentile met thresholds for excellent performance, defined as: 0.605–0.804 for Cohen’s Kappa and 0.795–0.894 for the remaining metrics (to three decimal places).

**Results:**

For balanced sample ratios, the optimum sample size for reliable models fell within the range of 750–1,000. Estimates increased to 1,100–1,300 for unbalanced samples with a 40:60 ratio of presence and absence data, respectively. Comparatively, unbalanced samples with a 20:80 ratio of presence and absence data did not produce reliable models with any of the sample sizes considered.

**Conclusion:**

To our knowledge, this is the first study to use a simulated vector to identify the optimum sample size for Random Forest models at this resolution (≤1 km^2^) and extent (≥10,000 km^2^). These results may improve the reliability of model predictions, optimise field sampling, and enhance vector surveillance in response to changing climates. Further research may seek to refine these estimates and confirm transferability to real vectors.

## 1 Introduction

Emerging infectious diseases have significantly increased, with vector-borne diseases (VBDs) accounting for 28.8% of emerging infectious events globally between 1990–2000 (1). VBDs have a detrimental impact on mortality, disability-adjusted life years, and economies (2–4). The World Health Organization (WHO) estimates that 80% of the world’s population are at risk of at least one VBD due to climate change, rapid urbanisation and globalisation (5). Across Europe, the incidence of endemic and (re-)emerging VBDs is changing. The warming of temperate regions has facilitated the latitudinal and altitudinal expansion of mosquitoes and ticks (6, 7). Cases of tick-borne encephalitis have increased, with six European countries considered highly endemic in 2020 (8); multiple regions reported West Nile virus for the first time in 2024 (9); and the invasive mosquito, *Aedes albopictus*, is firmly established in 14 European countries (10). Due to invasive mosquitoes, there have been 20 autochthonous dengue outbreaks, six autochthonous chikungunya outbreaks, and one autochthonous Zika outbreak in Europe since 2007 (11–13). Furthermore, it is projected that the invasive mosquitoes, *A. albopictus* and *Aedes aegypti*, will continue to expand into climatically suitable urban environments by 2050 (14).

**Figure 1:**
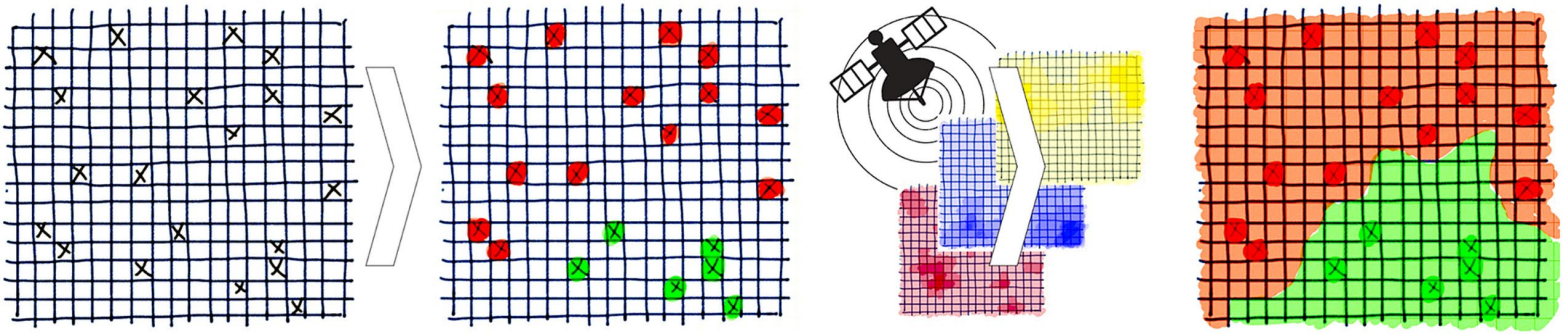
Overview of species distribution models. Random samples for vector distribution are linked to the environmental variables at each location and the model forms associations to generate a risk map based on the probability of vector presence across a region where sampling did not occur. Reprinted with permission (18).

To reduce the global incidence of VBDs by 60% from 2016 to 2030, the WHO recommends targeting the primary vectors (5). The WHO has provided a framework for effective vector control which builds on two foundational components: (1) enhanced capacity for surveillance, monitoring and evaluation and (2) increased research for vector control and innovation (5). The former was identified as a priority action for vector control in Europe (15). Field sampling monitors the distribution and abundance of vectors and is essential for surveillance, but is labour intensive and expensive (16). Species distribution models (SDMs) predict a region’s suitability for a species’ distribution under current or future eco-climatic conditions (17). This can optimise surveillance and reduce costs by identifying strategic locations for field sampling. As illustrated in Figure 1, correlative SDMs form associations between vector distribution and environmental variables to predict the probability of vector presence where sampling has not occurred (18). They achieve this by detecting patterns without explicitly defining biological processes, thus remaining independent of these assumptions for modelling (17). Within Europe, SDMs have successfully modelled the distribution of several arthropod vectors such as mosquitoes, sandflies and ticks (19–22).

SDMs are based on three main principles of spatial epidemiology: diseases tend to be limited geographically; the physical and biological conditions for vectors, hosts and pathogens influence the spatial heterogeneity of disease; and the current and future risks of disease are predictable if the abiotic and biotic factors can be delineated into maps (23). While it is possible to predict host and pathogen distributions, the drivers of pathogen transmission are complex, multifaceted and nonlinear (24). Furthermore, Hendrickx (25) used eco-climatic covariates to predict disease distribution in hosts and found that model accuracy decreased due to the increasing influence of other drivers, when compared to vectors. Therefore, modelling vector distributions as a proxy of VBD risk may be more appropriate. While vector presence does not necessarily infer a risk of disease, this approach aligns with the WHO and European Centre for Disease Prevention and Control (ECDC) recommendations for preparedness through vector surveillance (5, 26).

Ideally, SDMs are trained with presence and absence data since presence infers the locations which are environmentally suitable for a vector while absence infers the locations which are not (27). Vector distribution can be conceptualised as a gradient between potential distribution, which is where a species could live, and the realised distribution, which is where a species actually lives at a particular moment in time (28). Absence data is required to estimate the realised distribution, since distribution can be influenced by both abiotic and biotic factors (28). However, there is an inherent degree of uncertainty if an absence is a true absence since a species might be rare, in an inactive state, in a different habitat, or simply not captured by the trapping device (16). While absence data provides essential information, these limitations make field sampling more challenging, costly and labour-intensive to ensure their reliability (16). This has led to alternative techniques such as presence-only modelling and the generation of background samples or pseudo-absences. Background samples are generated computationally by randomly selecting points across a range of environmental conditions while pseudo-absences are manipulated to better represent a true absence (29). Several methods exist for generating pseudo-absences, such as excluding locations within a specific distance of a known presence point or comparing true absences for similar species (29). While pseudo-absences produce more accurate predictions than background samples, all three approaches have limitations: presence-only modelling fails to account for vector absence and the generation of absences makes significant assumptions which result in less accurate predictions compared to presence-absence models (29, 30). A simulated vector overcomes these limitations by generating high-quality presence and absences across different environmental gradients.

Various factors can influence model performance, including modelling algorithms, species characteristics, scale and sample size. Correlative SDMs can be developed with statistical or machine learning algorithms, but there is not a single best approach since each algorithm performs differently (31). The supervised machine learning algorithm, Random Forest (RF), can handle presence-absence data and thus model the vector’s realised distributions. Species range can impact model predictions since performance generally diminishes for species with broad geographic ranges and environmental tolerances compared to those with smaller ranges and specific tolerances (32). Scale can be divided into extent and resolution. Larger extents can improve model discrimination (28), but smaller extents can also enhance performance by reducing environmental variability (33). Resolutions larger than the species’ niche breadth typically decrease model accuracy (34). While sample size is also known to influence model predictions, SDMs have been developed with sample sizes ranging from 10 to over 1,000 (35). There is a lack of consensus between studies which evaluate the effect of sample size, but little distinction has been made between the use of different algorithms (32, 36–41), resolutions (33, 42, 43), ensembles (44) and species (45).

As part of a wider body of work, similar studies were identified which evaluated the effect of sample size [Supplementary material (1.2)]. Comparable studies were defined as RF models which predict the distribution of terrestrial species at similar resolutions (≤1 km^2^) and extents (≥10,000 km^2^). Two studies met this criteria and quantified the effect of sample size on RF models: Liu et al. (46) predicted the habitat suitability of the snail, *Oncomelania hupensis*, which is an intermediate host for *Schistosoma* spp. and Hendrickx et al. (18) predicted the distribution of the trematode, *Dicrocoelium dendriticum*, in ruminant hosts. Both used real datasets and neither considered sample sizes for vector distributions. Two studies used virtual species to analyse the effect of sample size at a similar scale, but they did not identify an optimum sample size and reported general trends instead (29, 47).

Despite increasing interest in SDMs, the conceptual and methodological uncertainties of these models are often overlooked (27). With a lack of standardisation, an increasing amount of freely accessible species distribution data and modelling software, Jiménez-Valverde et al. (28) argue that it is essential these uncertainties are addressed through the development of a solid methodological framework. A virtual vector facilitates the systematic evaluation of different methodological designs on model performance and, with a lack of consensus for the optimum sample size, there is a need to quantify the effect of sample size on model performance. Therefore, this empirical study aims to use a virtual vector to identify the optimum sample size for reliable large extent (≥10,000 km^2^) and fine resolution (≤1 km^2^) RF models.

## 2 Materials and methods

To evaluate the effect of sample size, two confounding factors were accounted for in this study: relative occurrence area (ROA) and prevalence. Jiménez-Valverde et al. (28) argue that the effect of species range on model performance is actually a reflection of the ROA which refers to the proportion of the test site occupied by the vector. If a species occupies a small area, then there are a greater number of absences located further away from vector presence which improves model discrimination (28). Therefore, this effect is scale dependent and independent of the species’ actual range size, since model performance can improve by using a larger extent which reduces the ROA (28). While the ROA is a function of extent, prevalence refers to the proportion of presence samples within the dataset and reflects the characteristics of the data (28). When compared to sample size and modelling technique, ROA and prevalence had the largest influence on model performance (44). Due to differing terminology between prevalence (28, 44), sample prevalence (38, 43) and presence prevalence (29), this study defined the proportion of presence and absence points as sample ratio. Sample size refers to the total number of presence and absence points in a sample. Both sample ratio and size refer to the dataset before partitioning, so that the results can guide the number of samples required during field sampling.

**Figure 2:**
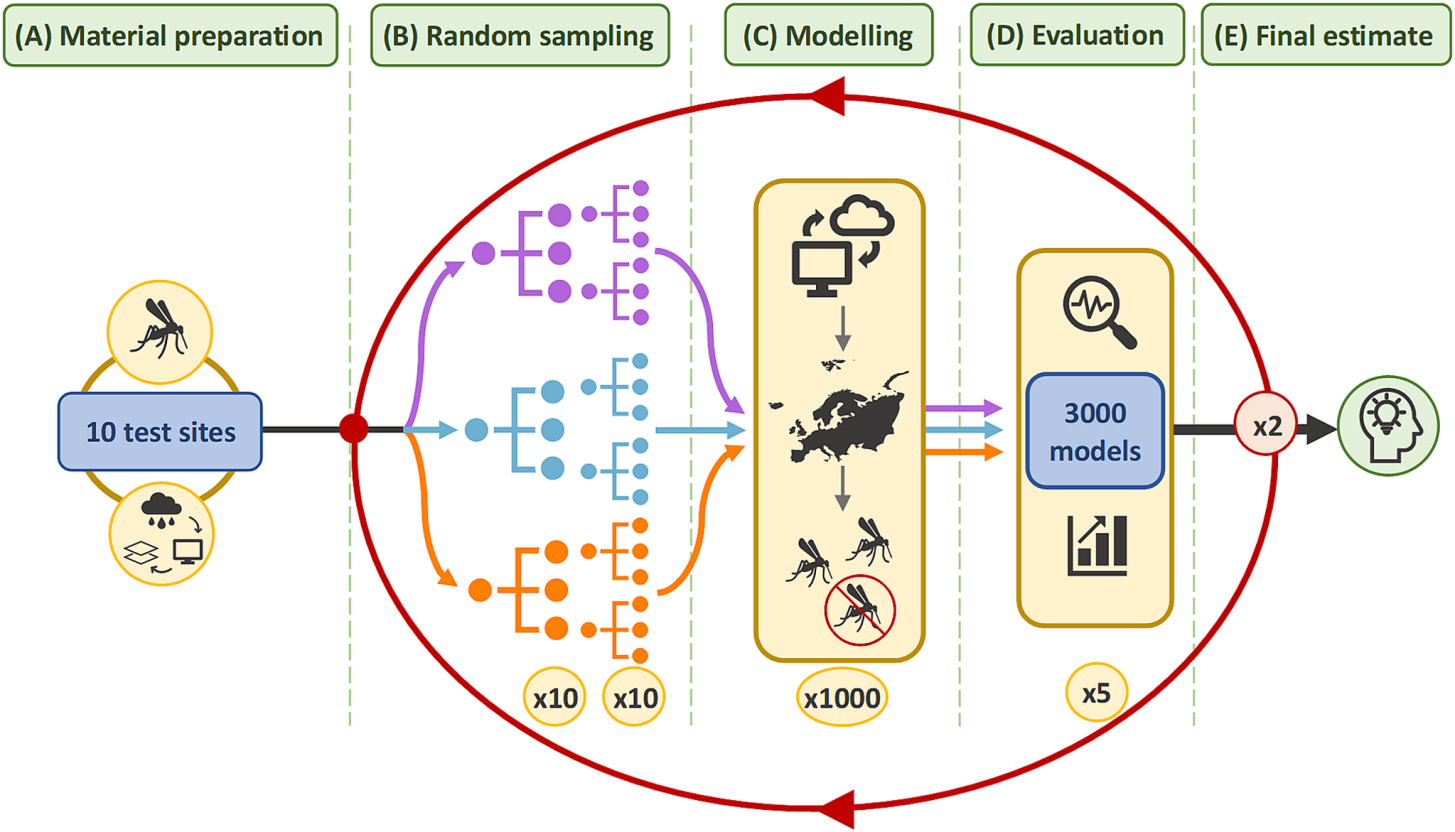
Methodology summary. In **(A)**, the virtual vector and covariate datasets were cropped to the extent of each test site. In **(B)**, 10 different sample sizes were randomly sampled from the virtual vector in each test site. Each sample size was replicated 10 times to produce 1,000 samples. This was repeated for three sample ratios. The coloured lines represent each sample ratio: purple for balanced samples with a 50:50 ratio; blue for samples with an unbalanced 20:80 ratio; and orange for samples with an unbalanced 40:60 ratio of presence and absence data, respectively. In **(C)**, 3,000 models were developed which predict the vector’s distribution in each test site. In **(D)**, the performance of 3,000 models was evaluated with five metrics. The results were grouped by sample size and sample ratio to identify a narrower range of sample sizes. In **(E)**, the methods from **(B–D)** were repeated twice more within increasingly narrower ranges to refine the estimate for the optimum sample size.

To account for ROA, the virtual vector was modelled in multiple test sites across Europe. Figure 2 illustrates a workflow whereby the effect of sample size was evaluated separately for three different sample ratios. Initially, 10 sample sizes were randomly generated. To account for variability in model performance, particularly at smaller sample sizes, sampling was replicated to generate 10 random samples, per sample size, in each test site. Model predictions were evaluated and performance grouped across the test sites. The methods were repeated twice more to refine estimates. Supplementary Figure 1 contains a flow chart detailing each step. The methodology was reported according to ODMAP, a standardised reporting protocol for SDMs (48) in Supplementary Table 1. The methods were executed in R Studio 2023.06.1 using R version 4.3.1 and the packages listed in Supplementary Table 2. QGIS 3.22.7 was used for figure generation and data quality checks.

### 2.1 Materials

For a species distribution model, datasets should describe the test site’s extent, the sampling location of each presence or absence record and the environmental conditions at each point. The vector distribution and covariate datasets need to have the same resolution (49). Therefore, all datasets were projected in the geographic coordinate reference system, EPSG:4326 – WGS 84 with matching spatial grids of 0.0083° × 0.0083°, which equates to approximately 1 km^2^ at the equator (0.918 km × 0.918 km to three decimal points).

#### 2.1.1 Virtual vector

**Figure 3:**
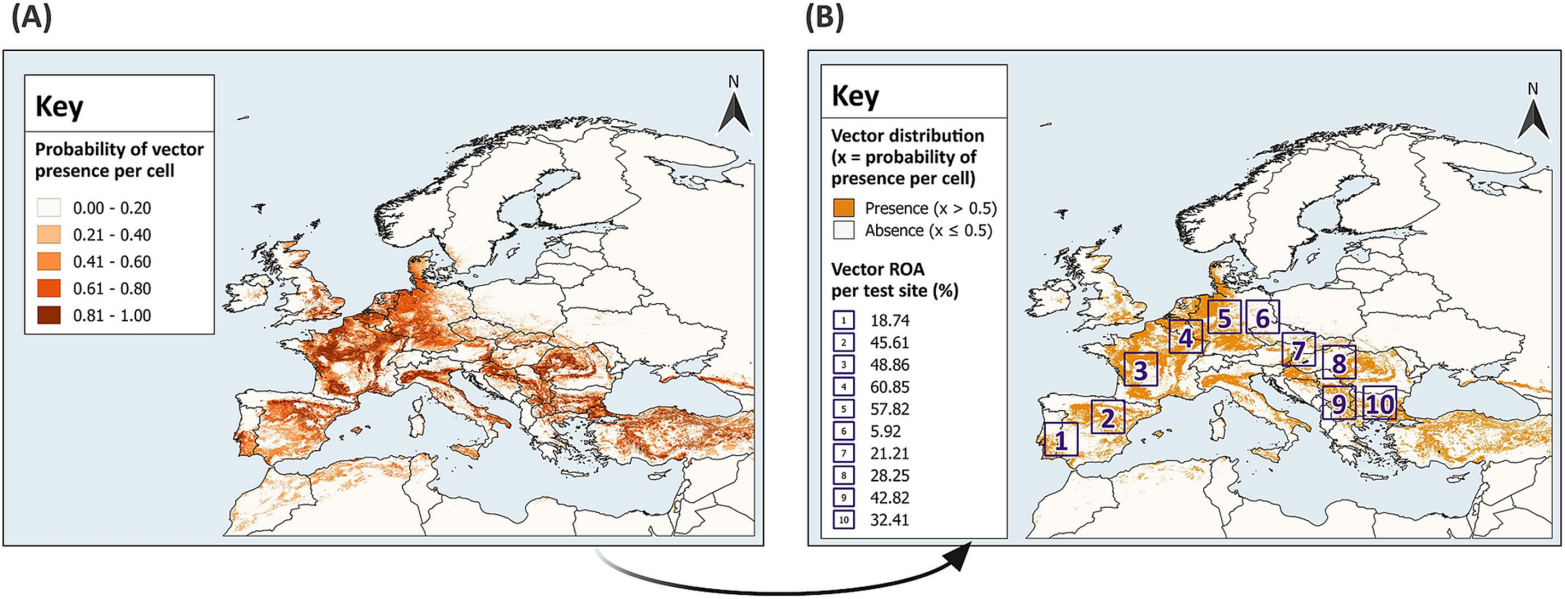
Distribution of the virtual vector across Europe. The virtual vector has a defined distribution within the extent of 13.0°W – 43.0°E, 29.0°N – 72.0°N **(A)**, which was classified into binary values for presence and absence using a threshold of 0.5 **(B)**. In **(A)**, a gradient from light to dark represents increasing probabilities of presence per raster cell, categorised into five classes using equal intervals. In **(B)**, sampling and modelling was conducted in 10 test sites across Europe. The Relative Occurrence Area (ROA) of the vector was defined as the percentage of cells with a presence value of 1, divided by the total number of cells within each test site (to two decimal points). GADM Level 0 country boundaries were utilised (79).

SDMs should be developed with high quality, fine resolution datasets for vector presence and absence with georeferenced locations that are spatially and environmentally unbiased, but this is difficult to attain (37). With a known distribution and presence-absence data, the virtual vector enables the prediction of realised distributions, overcoming challenges associated with historical and field datasets for real vectors. The virtual vector was generated using the probability approach from the SDMvspecies package in R (50). This method is preferable to the threshold approach since it mimics vector occupancy patterns across space and time by generating species distributions across environmental gradients and takes species prevalence into consideration (37). The virtual vector was modelled to resemble a flying insect and has a fully known spatial distribution across Europe (Figure 3A). For direct comparison between the predicted and known distribution, the virtual vector was classified into binary values; a threshold of 0.5 was applied, and cells with a probability of presence greater than 0.5 were assigned a value of 1 for presence, while cells with a probability of presence equal to or less than 0.5 were assigned a value of 0 for absence (Figure 3B).

Random sampling and modelling were conducted within 10 test sites across mainland Europe (Figure 3B). Modelling is often tailored to the research objective. To estimate the optimum sample size and provide generalised guidance for field sampling and subsequent modelling, test sites with differing characteristics were selected. Each reflects varying geographic distributions of the virtual vector; thus, grouping model performance by sample size across the test sites accounts for differing habitat suitability and ROA. The test sites have an extent comparable to some European countries with an area of 11.111°^2^, which equates to approximately 136,900 km^2^ at the equator (370 km × 370 km). The virtual vector dataset was cropped to the extent of each test site to create 10 distinct datasets for sampling and modelling.

#### 2.1.2 Covariates

Environmental variables typically represent the key aspects of a species’ ecology which impact its survival in a particular environment (51). Vectors are ectothermic and while the degree of impact is species specific, temperature is one of the main environmental factors which can affect their reproduction, survival, distribution and ability to transmit pathogens (52). Environmental variables like vegetation parameters can also impact vector presence (6, 52). Remotely sensed covariates from satellites may be preferable to climatic covariates due to their high spatial and temporal resolution globally (53). Therefore, time series data for 27 covariates which describe normalised difference vegetation index (NDVI) and land surface temperature (LST) from 2001 to 2021 were obtained from MODerate-resolution Imaging Spectroradiometer (MODIS) satellite imagery (Table 1). Following methods by Scharlemann et al. (54), the covariates were temporal Fourier processed, thereby reducing data dimensionality and removing correlations to create independent covariates. The covariate dataset was then cropped to the extent of each test site.

**Table 1:** Covariates used for modelling.

Covariate	Description
dLSTa1	Daytime Land Surface Temperature annual amplitude (K)
dLSTa2	Daytime Land Surface Temperature bi-annual amplitude (K)
dLSTa3	Daytime Land Surface Temperature tri-annual amplitude (K)
dLSTavg	Mean daytime Land Surface Temperature (K)
dLSTmax	Maximum daytime Land Surface Temperature (K)
dLSTmin	Minimum daytime Land Surface Temperature (K)
dLSTp1	Daytime Land Surface Temperature phase of annual cycle (months)
dLSTp2	Daytime Land Surface Temperature phase of bi-annual cycle (months)
dLSTp3	Daytime Land Surface Temperature phase of tri-annual cycle (months)
NDVIa1	Normalised Difference Vegetation Index annual amplitude (no units*)
NDVIa2	Normalised Difference Vegetation Index bi-annual amplitude (no units*)
NDVIa3	Normalised Difference Vegetation Index tri-annual amplitude (no units*)
NDVIavg	Mean Normalised Difference Vegetation Index (no units*)
NDVImax	Maximum Normalised Difference Vegetation Index (no units*)
NDVImin	Minimum Normalised Difference Vegetation Index (no units*)
NDVIp1	Normalised Difference Vegetation Index phase of annual cycle (months)
NDVIp2	Normalised Difference Vegetation Index phase of bi-annual cycle (months)
NDVIp3	Normalised Difference Vegetation Index phase of tri-annual cycle (months)
nLSTa1	Night-time Land Surface Temperature annual amplitude (K)
nLSTa2	Night-time Land Surface Temperature bi-annual amplitude (K)
nLSTa3	Night-time Land Surface Temperature tri-annual amplitude (K)
nLSTavg	Mean night-time Land Surface Temperature (K)
nLSTmax	Maximum night-time Land Surface Temperature (K)
nLSTmin	Minimum night-time Land Surface Temperature (K)
nLSTp1	Night-time Land Surface Temperature phase of annual cycle (months)
nLSTp2	Night-time Land Surface Temperature phase of bi-annual cycle (months)
nLSTp3	Night-time Land Surface Temperature phase of tri-annual cycle (months)

All files were created by transforming MODIS historical climate data, from 2001–2021, for daytime Land Surface Temperature (dLST), night-time Land Surface Temperature (nLST) and Normalised Difference Vegetation Index (NDVI) via temporal Fourier analysis. As described by Scharlemann et al. (54), the NDVI covariates denoted by the symbol * do not have units because they are dimensionless ratios and therefore, categorical. The international base unit for temperature, Kelvin, is denoted by K.

### 2.2 Methods

#### 2.2.1 Sampling

**Figure 4:**
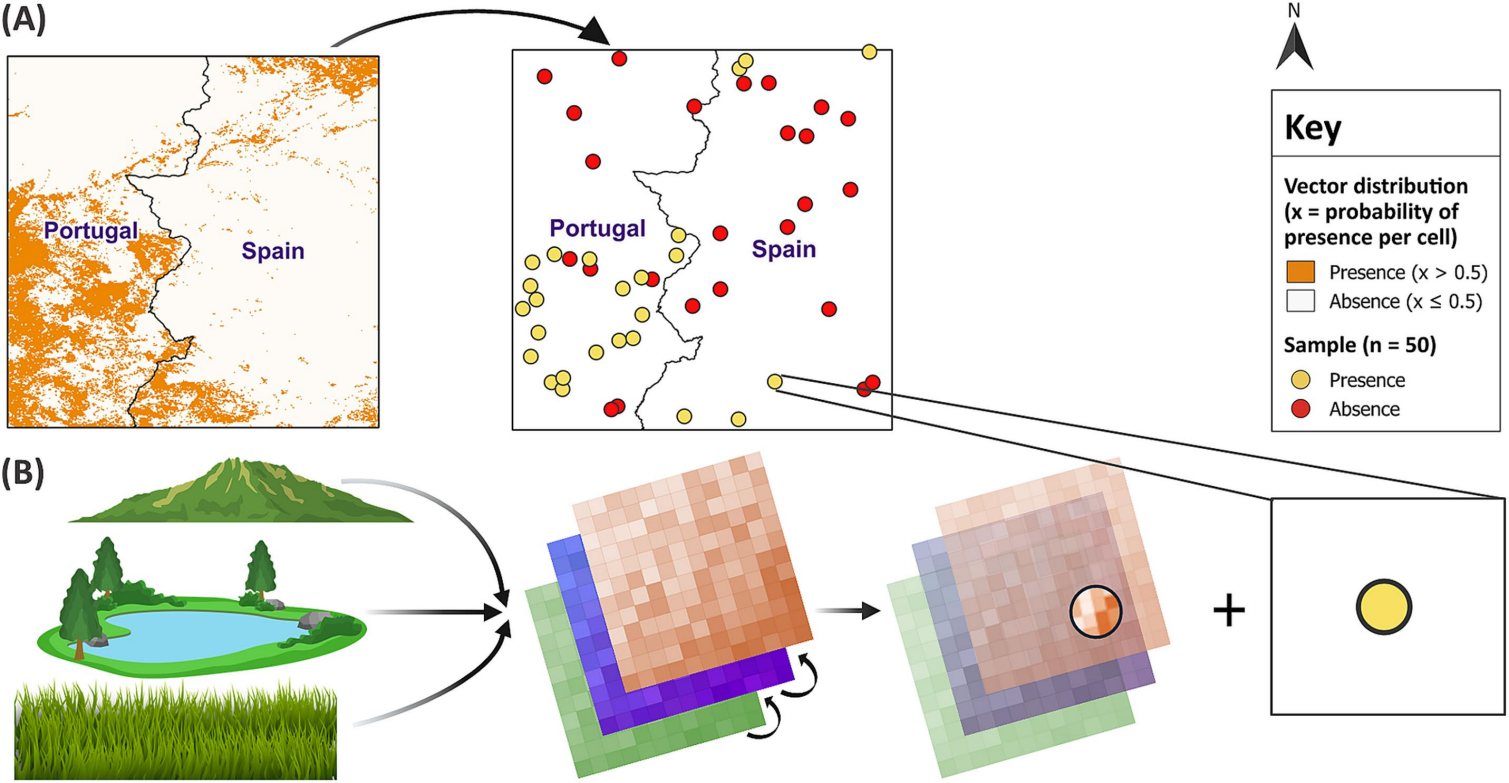
Random sampling and linkage of datasets. In **(A)** random sampling of the binary virtual vector in test site 1 generated a balanced sample size of 50. In **(B)** each presence and absence point was linked to the covariates at that location. GADM Level 0 country boundaries were utilised (79).

First, 10 different sample sizes (10, 30, 50, 80, 100, 250, 500, 1,000, 2,500 and 5,000) were randomly sampled from the virtual vector’s distribution in each of the 10 test sites. Random sampling reduces the likelihood of oversampling a particular area. Geographically clustered samples can decrease predictive accuracy and may have a larger influence than sample size (18). To minimise spurious results, each sample size was replicated 10 times in each test site, generating 1,000 unique samples. Since the proportion of presence observations in a sample has an important influence on model performance (43, 44), the methods were repeated for three sample ratios with different proportions of presence and absence observations. These included a balanced 50:50 ratio and two unbalanced ratios of presence and absence points (40:60 and 20:80, respectively). Given the increased probability of sampling absences compared to presences in the field, the unbalanced sample ratios contained a greater proportion of absence points. This approach was intended to represent field conditions for vector sampling. For each of the 3,000 samples, the presence and absence points were linked to the covariates describing the environmental conditions at each location (Figure 4).

#### 2.2.2 Modelling

**Figure 5:**
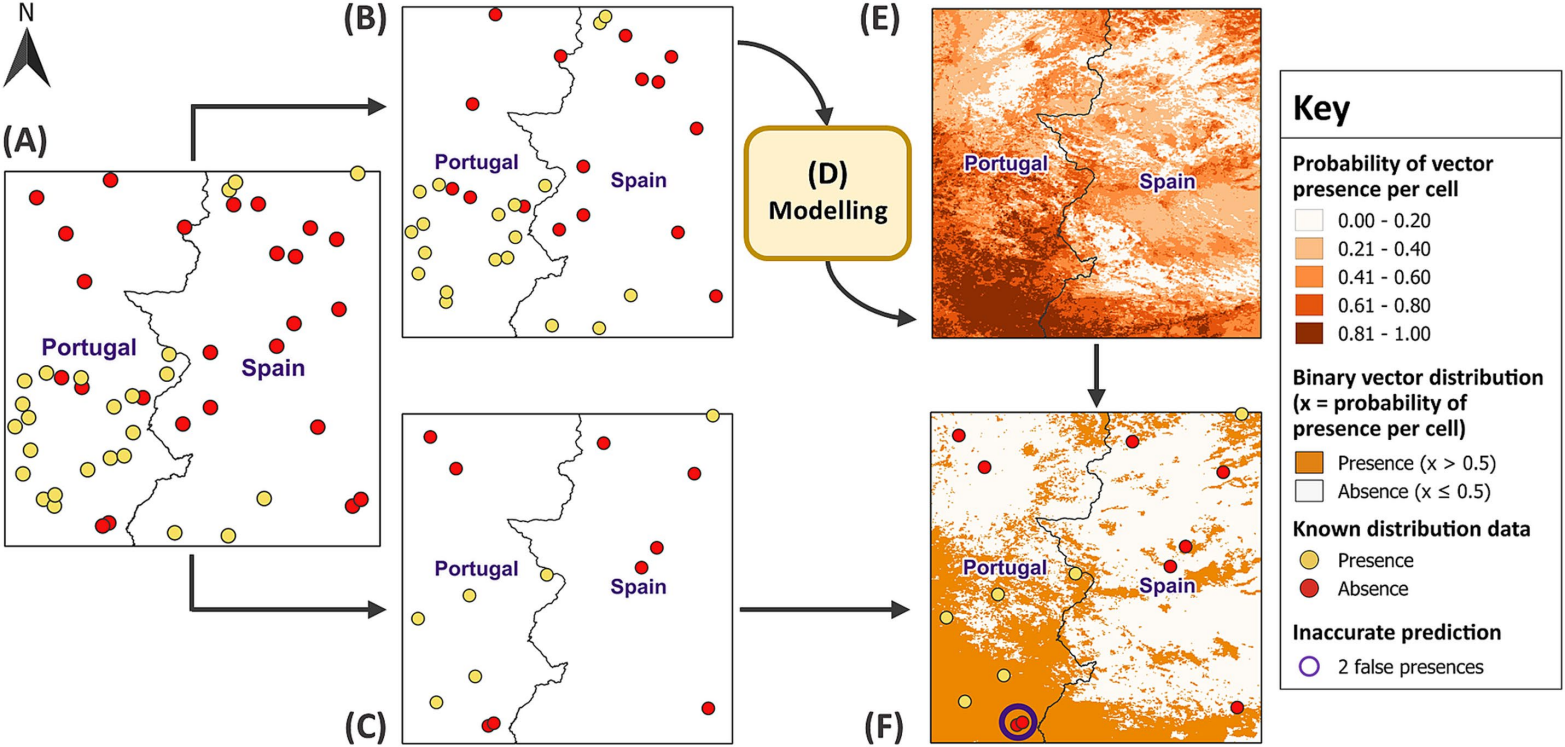
Training models and predicting vector distribution. In **(A)** a balanced sample size of 50 was randomly partitioned into **(B)** a training subset (70% of the sample) and **(C)** a test subset (30% of the sample). In **(D)** the model was developed using the training subset before the covariates for test site 1 were applied to **(E)** predict vector distribution across the remainder of the test site. The model predictions represent increasing probabilities of vector presence per raster cell, categorised into five classes using equal intervals. In **(F)**, the predictions were dichotomised into binary values for presence and absence using a threshold of 0.5. The test subset validated the model predictions, revealing two locations, circled in purple, where the model predicted presence, but the vector was actually absent. GADM Level 0 country boundaries were utilised (79).

RF has recently gained popularity due to its ease of use and ability to model non-linear relationships and complex interactions between covariates (29). RF can use classification or regression trees for binary data. However, regression RF has been shown to predict the probabilities of species distribution with greater accuracy (55). Therefore, each sample trained a regression RF model which predicted the virtual vector’s distribution in its respective test site. To do so, the sample was randomly split into a test and training subset using a 30:70 ratio, respectively (Figure 5). This is a cross-validation method which uses a subset of data to train the model before comparing the predictions with the vector’s known distribution from the test subset. Since RF creates robust models with the default parameters (56), the only specified parameters were the hyperparameter *mtry* (optimum) and the number of trees (500). A higher number of trees is recommended to improve the accuracy of model predictions, but this needs to be balanced against computational cost (56). Once 3,000 models formed associations between vector distribution and covariates from each training subset, the models used the covariates to make predictions for vector distribution across their respective test site. For evaluation, the predictions were dichotomised into binary values for presence (1) and absence (0) using the same threshold of 0.5. Each presence and absence point in the independent test subsets were then linked to their corresponding model predictions.

#### 2.2.3 Evaluation

Model performance was assessed using five metrics: Percentage Correctly Classified (PCC), sensitivity, specificity, Cohen’s Kappa and Area Under the Curve (AUC). PCC, sensitivity, specificity and Cohen’s Kappa are threshold-dependent metrics, calculated from confusion matrices comparing the binary model predictions for vector presence and absence against the virtual vector’s known distribution (Table 2). PCC measures the proportion of correctly predicted presence and absence observations; sensitivity measures the proportion of correctly predicted presence observations; and specificity measures the proportion of correctly predicted absence observations in the test subset (57). The values for all three metrics range from 0 to 1, with a higher value indicating better model performance. Unlike the previous metrics which solely calculate the proportion of agreement between model predictions and known observations, Cohen’s Kappa also accounts for chance agreement when calculating inter-rater reliability (58, 59). The values for Cohen’s Kappa range from −1 to 1, whereby 1 indicates perfect agreement between known and predicted observations while values equal to or less than 0 suggest the model’s performance is no better than chance (58). Each of these metrics were cross-referenced against R functions: pcc from the PresenceAbsence package, sensitivity and specificity from the caret package, and the unweighted value for Kappa from the vcd package (60–62).

**Table 2:** Calculations for four threshold-dependent evaluation metrics.

Confusion matrix		
	Actual presence	Actual absence
Predicted presence	A	B
Predicted absence	C	D

Evaluation metrics	
Sum	n=(A+B+C+D)
PCC	$\frac{A+D}{n}$
Sensitivity	$\frac{A}{\left(A+C\right)}$
Specificity	$\frac{D}{\left(B+D\right)}$
Cohen’s Kappa	$\text{Cohen}\prime s\;\text{Kappa}=\frac{P_{o}-P_{e}}{1-P_{e}}$, whereby:$\text{Proportion of observed agreements}\;(P_{o})=\sum P_{\text{ii}}\;$, equates to: $P_{o}=\frac{\left(A+D\right)}{n}$ $\text{Proportion of chance agreement}\;(P_{e})=\sum P_{i}+P_{+i}$, equates to:$P_{e}=((\frac{A+B}{n})\times (\frac{A+C}{n}))+((\frac{C+D}{n})\times (\frac{B+D}{n}))$

Each of the threshold-dependent evaluation metrics were based on a 2×2 contingency table for model performance which compared the binary model predictions to the actual presence and absence points in the test subset. The counts describe when the model (A) correctly predicts presence (true positives), (B) incorrectly predicts presence at a location where absence was actually recorded (false positives), (C) incorrectly predicts absence at a location where presence was actually recorded (false negatives), and (D) correctly predicts absence (true negatives).

AUC, a threshold-independent metric, is derived from the receiver operating curve which analyses how different thresholds influence the classification of presence and absence by plotting sensitivity on the y-axis against (1 – specificity) on the x-axis (63). As such, AUC provides a summary of classification accuracy with values ranging from 0–1, whereby 1 indicates perfect accuracy and 0.5 suggests the model predictions are equivalent to random chance (63). AUC was calculated using the R functions, roc and auc from the pROC package (64). All five metrics are commonly used to evaluate SDMs and, as recommended by Konowalik and Nosol (65), the use of multiple metrics overcomes the limitations associated with relying on a single metric.

#### 2.2.4 Optimum sample size

For each of the five evaluation metrics, the 3,000 models were grouped by sample size and ratio across the 10 test sites and presented in boxplots. Model performance was assessed against predefined thresholds, which determine excellent model performance for each evaluation metric (Table 3). The thresholds for Cohen’s Kappa and AUC were based on those presented by Landis and Koch (66) and Hosmer et al. (67), respectively. Expert advice informed the thresholds for PCC, sensitivity and specificity. The thresholds for excellent performance were defined as: 0.795–0.894 for PCC, sensitivity, specificity, and AUC; and 0.605–0.804 for Cohen’s Kappa (to three decimal places). To identify the optimum sample size, the boxplots were examined to identify at what sample size the 25th percentile (first quartile of each boxplot) met these thresholds.

**Table 3:** Thresholds for excellent model performance for each evaluation metric.

PCC, Sensitivity and Specificity	Cohen’s Kappa	AUC	Rating
N/A	< 0.00	≤ 0.50	Chance
0.00–0.49	0.00–0.20	0.51–0.69	Poor
0.50–0.69	0.21–0.40	N/A	Fair
0.70–0.79	0.41–0.60	0.70–0.79	Moderate
0.80–0.89	0.61–0.80	0.80–0.89	Excellent
0.90–1.00	0.81–1.00	0.90–1.00	Outstanding

To refine estimates for the optimum sample size, these methods were repeated twice more within increasingly narrower ranges of sample sizes, generating 9,000 models. For simplification, all 9,000 models were then grouped by sample size and ratio and presented in heatmaps. The 25th percentile for each sample size and ratio was calculated, and the same thresholds for excellent performance applied to identify the optimum sample size. Moderate performance was also taken into consideration, defined as: 0.695–0.794 for PCC, sensitivity, specificity, and AUC; and 0.405–0.604 for Cohen’s Kappa (to three decimal places).

## 3 Results

The first round of modelling evaluated the effect of sample sizes 10, 30, 50, 80, 100, 250, 500, 1,000, 2,500, and 5,000 on model performance. Boxplots for each evaluation metric were compared against the thresholds for excellent model performance, which indicated that the optimum sample size fell within the range of 250–2,500 (Supplementary Figure 2). To include a margin of error, the range was adjusted to 150–3,000, from which 10 sample sizes were selected. The methods were repeated with the sample sizes 150, 250, 350, 500, 750, 1,000, 1,500, 2,000, 2,500, and 3,000, identifying a narrower range between 400–1,300 (Supplementary Figure 3). The results for the final round, which evaluated the effect of sample sizes 400, 500, 600, 700, 800, 900, 1,000, 1,100, 1,200, and 1,300 on model performance, are presented below.

### 3.1 Model performance

**Figure 6:**
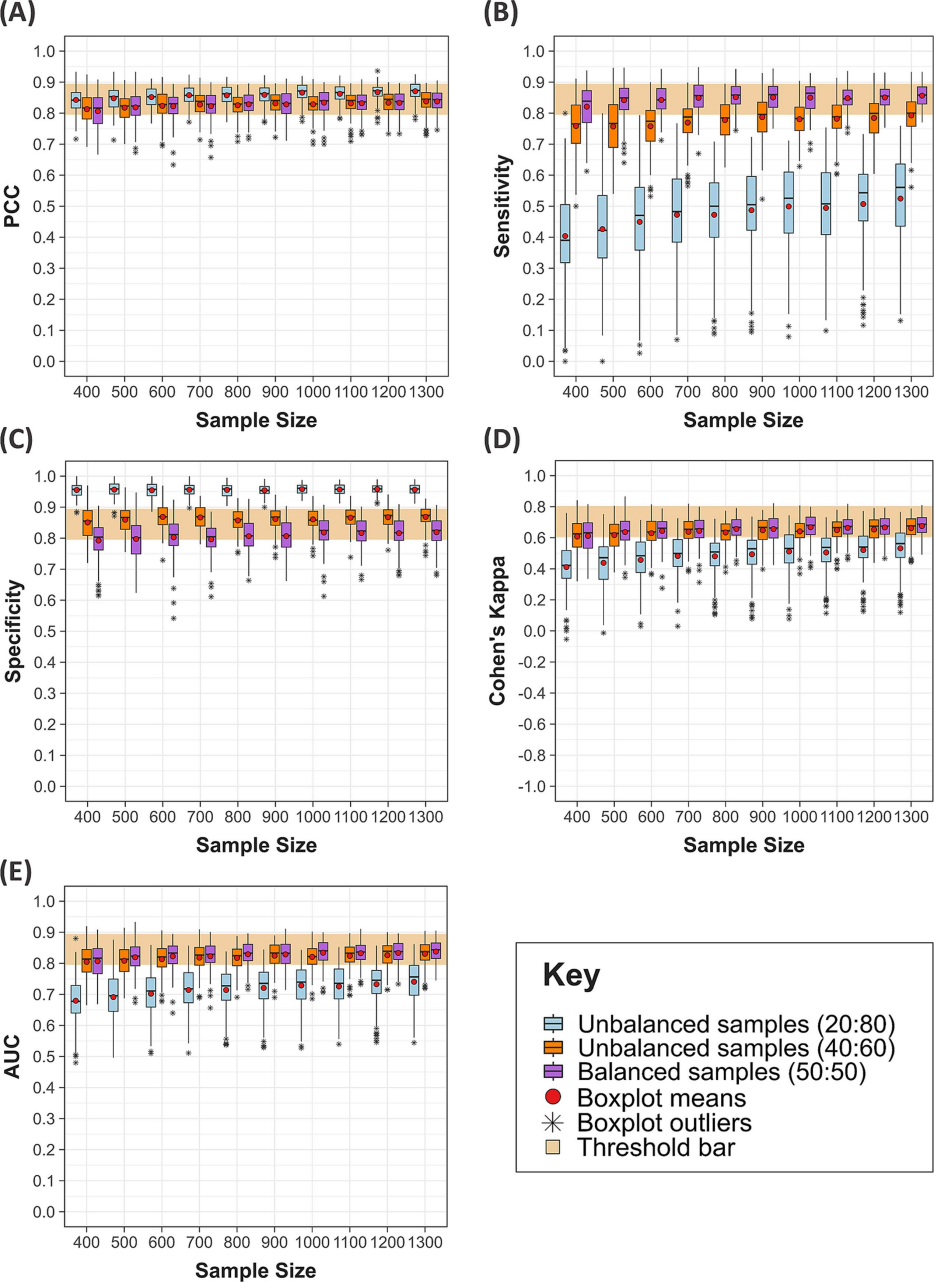
Performance of 3,000 models when evaluated by five metrics. Each boxplot represents the means, medians and quartiles of 100 models which are grouped by the 10 sample sizes (400, 500, 600, 700, 800, 900, 1,000, 1,100, 1,200, and 1,300) and three sample ratios of presence and absence data (50:50, 20:80 and 40:60, respectively). The threshold bar represents the defined thresholds for excellent models (to three decimal points): 0.795–0.894 for **(A–C,E)** which present the PCC, sensitivity, specificity and AUC metrics, respectively, and 0.605–0.804 in **(D)** which presents Cohen’s Kappa.

Increasing sample size improved model performance across all sample ratios and evaluation metrics (Figure 6). The sample size which first reached the thresholds for excellent model performance varied between sample ratios and metrics. For balanced ratios, the first sample size to reach the thresholds was 500, when evaluated by sensitivity (first quartile = 0.811, mean = 0.842), compared to a sample size of 1,000, when evaluated by specificity (first quartile = 0.795, mean = 0.818). The smallest sample size to reach the thresholds for unbalanced 40:60 ratios was 400, when evaluated by specificity (first quartile = 0.809, mean = 0.851), and the largest was 1,100, when evaluated by Cohen’s Kappa (first quartile = 0.608, mean = 0.649). Comparatively, for unbalanced 20:80 ratios, no sample size met the thresholds for sensitivity, Cohen’s Kappa or AUC, while all reached or exceeded the PCC and specificity threshold. For three out of five metrics (sensitivity, Cohen’s Kappa and AUC), models developed with balanced sample ratios were more reliable than those developed with unbalanced ratios.

### 3.2 Model performance across all sample sizes

**Figure 7:**
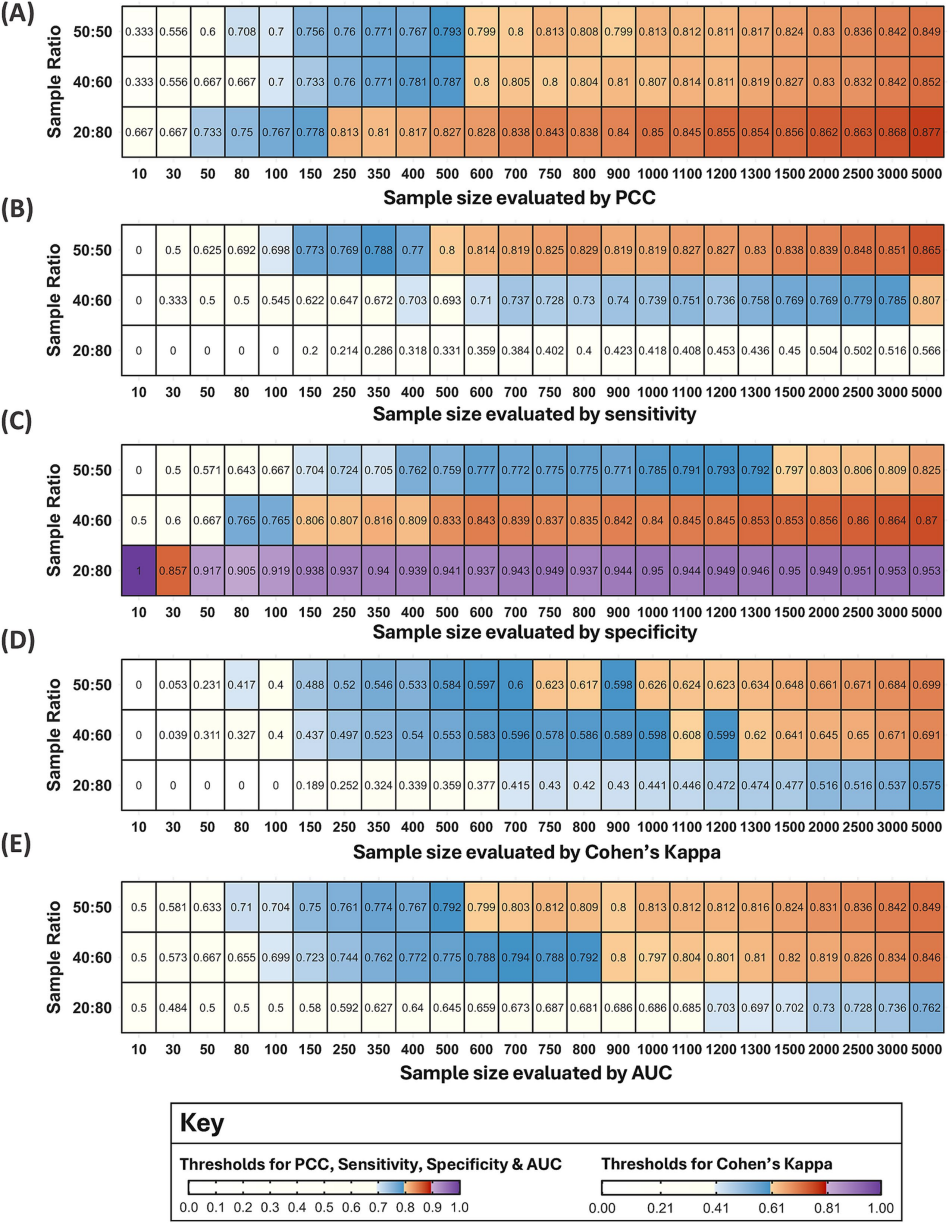
Heatmaps for five metrics evaluating the performance of 8,999 models. Each tile shows the value of the first quartile for each sample size and ratio. Note: One model (out of 9,000) failed for a sample size of 10 with a 20:80 ratio. Therefore, some tiles may represent more than 100 models if the same sample size was evaluated in multiple rounds, or fewer if a metric could not be calculated. The colour gradients from light to dark represent increasing performance within the thresholds for moderate (blue) and excellent models (orange). For **(A–C,E)** moderate performance was defined as 0.695–0.794 to three decimal points and excellent performance at 0.795–0.894 for PCC, sensitivity, specificity, and AUC, respectively. For Cohen’s Kappa in **(D)** moderate performance was defined as 0.405–0.604 and excellent performance as 0.605–0.804. Performance below the moderate thresholds was coloured white and above the excellent thresholds, purple.

A total of 8,999 models were developed with 24 different sample sizes, some of which were evaluated multiple times (250 and 500 twice; 500 and 1,000 three times). Models were grouped by sample size and ratio and presented as heatmaps for each metric (Figure 7). A blue and orange gradient from light to dark indicates how far the first quartile lies within the thresholds for moderate and excellent performance, respectively.

Model performance notably deteriorated for sample sizes below 100 across all metrics, with few reaching the thresholds for moderate performance (Figure 7). One model developed with a sample size of 10 and a 20:80 ratio failed, and 93 models with the smallest sample sizes (10 and 30) had at least one incalculable metric due to a lack of presence or absence points in the test and/or training subsets. Of the 93 models with missing metrics, 60% were developed with a 20:80 ratio (56/93), 21% with a 50:50 ratio (20/93), and 19% with a 40:60 ratio (18/93). Therefore, RF models may produce unreliable predictions for vector distribution when developed with a sample size of 10 and 30, particularly with an unbalanced 20:80 ratio. Model performance for this ratio also varied substantially across the metrics at other sample sizes, indicating that model predictions with a 20:80 ratio were the least reliable.

The first sample sizes to reach each metric’s thresholds varied between 400–1,100 for unbalanced 40:60 ratios and between 500–1,000 for balanced 50:50 ratios in Figure 6. However, these findings do not account for all sample sizes considered or varying model performance for the same sample sizes evaluated in multiple rounds. For balanced ratios, a sample size of 1,000 first reached the specificity threshold in the third round (first quartile = 0.795, mean = 0.818), but the same sample size did not meet the threshold in the first round (first quartile = 0.793, mean = 0.818) or the second round of methods (first quartile = 0.781, mean = 0.813). Figure 7 suggests the first sample size to reach each metric’s thresholds varied between 150–5,000 for unbalanced 40:60 ratios and between 500–1,500 for balanced 50:50 ratios.

Important trends may be overlooked when solely focusing on the first sample size to reach thresholds for excellent model performance. For an unbalanced 40:60 sample ratio, the first quartile reached the PCC threshold for excellent model performance from a sample size of 600. There were minor fluctuations of 0.01 decimal places around the threshold boundary until a sample size of 1,100, after which performance consistently improved with increasing sample size (Figure 7A). For sensitivity, the first quartile only reached the threshold for excellent performance at a sample size of 5,000. Since the models were trained with a greater proportion of absence data, it may be reasonable to consider that the mean reached the threshold for excellent performance at a sample size of 1,500 (mean = 0.799) and the first quartile for a sample size of 1,100 reached the upper estimate (above 0.75) for moderate performance (Figure 7B). For specificity, the first quartile reached the threshold for excellent performance from a sample size of 150. Due to minor fluctuations at the boundary, performance was more reliably above the threshold from a sample size of 500 (Figure 7C). When evaluated by Cohen’s Kappa, a sample size of 1,100 met the threshold for excellent performance, but dipped below at 1,200, suggesting models may be more reliable from 1,300 (Figure 7D). Finally, a sample size of 900 reached the AUC threshold for excellent models with fluctuations around the boundary until a sample size of 1,300 (Figure 7E).

For balanced ratios, the first quartile reached the PCC and AUC threshold for excellent performance at a sample size of 600 but remained near the boundary until a sample size of 1,000 (Figures 7A,E). For sensitivity, a sample size of 500 first reached the threshold but models were more comfortably above the threshold from a sample size of 600. However, there was a slight dip at a sample size of 900 and 1,000, albeit by 0.01 decimal places, after which performance continuously improved with increasing sample size (Figure 7B). For specificity, the first quartile reached the thresholds for excellent models from a sample size of 1,500, but performance was only 0.01 decimal places below the threshold from a sample size of 1,000 (Figure 7C). For Cohen’s Kappa, the first quartile reached the threshold for excellent performance at a sample size of 750 but dipped below at a sample size of 900, suggesting models may be more reliable from a sample size of 1,000 (Figure 7D).

### 3.3 Estimates for the optimum sample size

The optimum sample size was estimated separately for the three sample ratios. To determine the optimum sample size, more emphasis was placed on sensitivity, Cohen’s Kappa and AUC. While PCC is a widely used metric, it is a poor reflection of model performance since it is influenced by the ROA: if the vector occupies 5% of the test site, a 95% success rate could be achieved if the model predicted absence across the entire test site (68). Since specificity measures the percentage of absence observations correctly predicted, this should theoretically increase when models are trained on a greater proportion of absence points which explains why the 20:80 sample ratio performed best when evaluated by specificity. RF models also tend to overfit resulting in higher sensitivity and lower specificity values (44). However, it may be preferable to maximise sensitivity over specificity when the aim is to inform new vector surveys, since higher sensitivity minimises the number of true presences predicted as absences (69).

**Figure 8:**
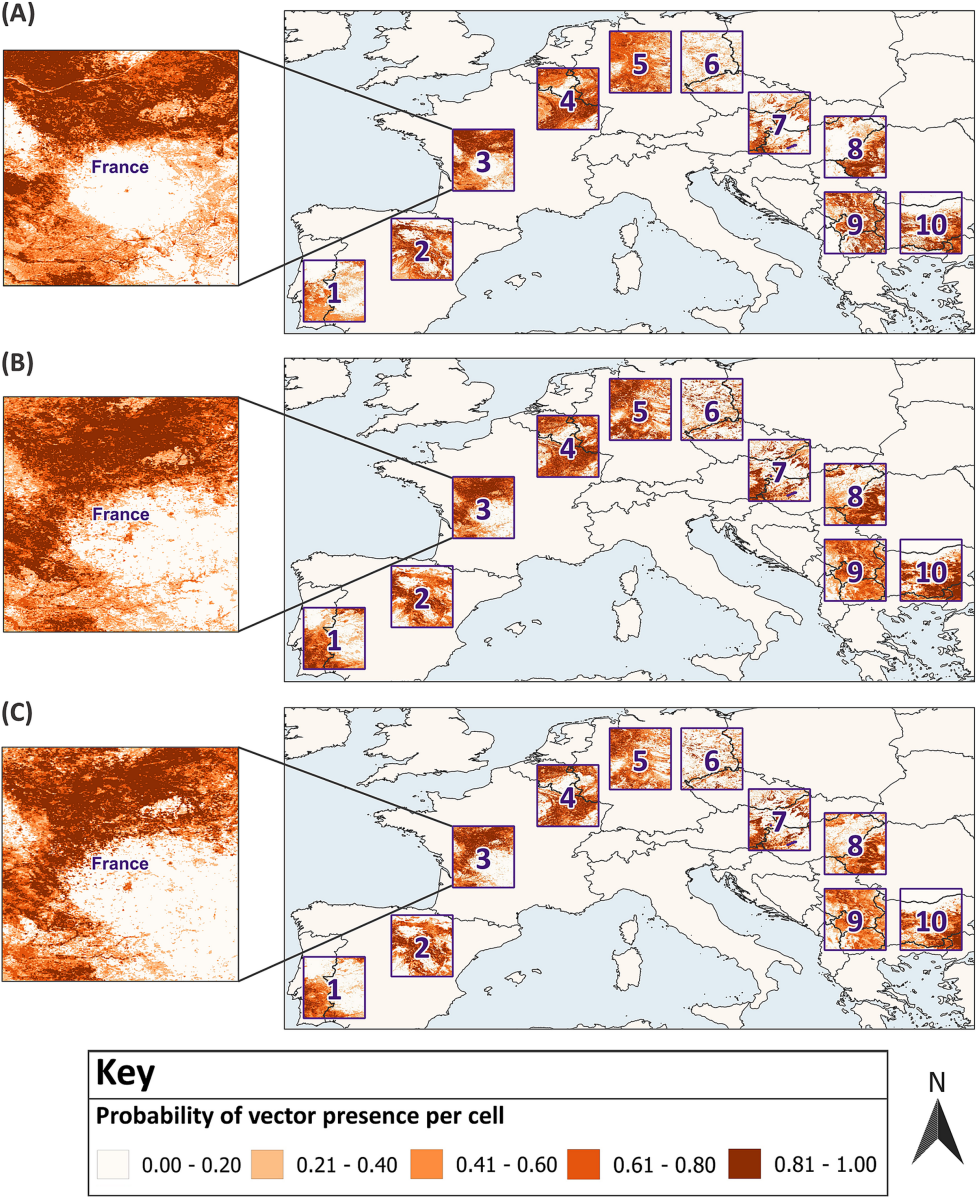
Model predictions developed with the optimum sample size compared to the virtual vector’s known distribution. In **(A)** the actual distribution of the virtual vector in 10 test sites across Europe is displayed against one model’s predictions which were developed with **(B)** a balanced 50:50 sample ratio and a sample size of 750 and **(C)** an unbalanced 40:60 sample ratio and a sample size of 1,100. Each map displays increasing probabilities of presence per raster cell, categorised into five classes using equal intervals. GADM Level 0 country boundaries were utilised (79).

The optimum sample size was best expressed as a range since there were fluctuations around the threshold boundaries for excellent model performance between sample sizes and for the same sample size across multiple rounds. Most metrics indicated that the optimum sample size fell between the range of 750–1,000 for balanced ratios. This increased to 1,100–1,300 for an unbalanced 40:60 sample ratio of presence and absence data, respectively. Due to poor model performance, it was not possible to estimate an optimum sample size for a 20:80 ratio. Model predictions for vector distribution, developed with the lower estimate for the optimum sample size, are displayed across all 10 test sites (Figure 8). This illustrates one model’s predictions with the optimum sample size, however stacked predictions should be considered due to the variation between replicate models at the same sample size (Figure 6). Additional figures which spatially present predicted vector distributions by models with different sample sizes are available in Supplementary Figures 4–9.

## 4 Discussion

To the best of our knowledge, this is the first study to use a virtual vector to identify the optimum sample size for RF models with presence-absence data at this extent (≥10,000 km^2^) and resolution (≤1 km^2^). This study produced three main findings; the optimum sample size for reliable SDMs fell within the range of 750–1,000 for balanced samples and 1,100–1,300 for samples with an unbalanced 40:60 ratio of presence and absence points, respectively. Secondly, model performance was poor for sample sizes below 100 and for samples with an unbalanced 20:80 ratio. Thirdly, as the proportion of presence points increased between sample ratios, model performance improved for all metrics except PCC and specificity.

### 4.1 Sample size for Random Forest models

An optimum sample size facilitates robust predictions for vector distributions while minimising the cost of excessive field sampling and computational processing. Many studies have demonstrated improvements in RF model performance with increasing sample size (18, 29, 33, 42–47). However, it is important to consider that these studies used different definitions for sample size when comparing results. Sample size was defined as either the number of presences in the training subset (18, 29, 33, 42, 43, 47) or the number of presences and absences in the training subset (44–46). To ensure our findings could inform field sampling, we defined sample size as the number of presences and absences in the dataset, prior to partitioning.

Our estimates for the optimum sample size were lower than two studies which quantified the effect of sample size on RF models at a similar scale (18, 46). Liu et al. (46) used field data for an intermediate host and reported an optimum sample size of 2,400 presence and absence points in the training subset, with an optimum sample ratio of 1:2 (equivalent to 33:66). Since they used an 80:20 ratio to partition their samples into training and test subsets respectively, their estimate equates to 3,000 presence and absence points overall. Their sample ratio is closest to the 40:60 ratio considered in this study, but their estimates are nearly three times greater than our optimum sample size of 1,100–1,300. However, their models also performed better at smaller sample sizes. A sample size of 100 with their optimum ratio (first quartile = 0.894, median = 0.917) far exceeded our AUC thresholds for excellent performance (46), which differs from our findings. Since virtual vectors are a simplification of reality, estimates for the optimum sample size are expected to increase since real host and vector distributions have more complex responses to covariates (38). However, differing methodology is the most likely reason for the different estimates for the optimum sample size. Liu et al. (46) determined the optimum sample size by identifying a point at which there was a significantly small increase in AUC and sensitivity began to decrease.

On the other hand, improvements in model performance at small sample sizes likely reflect different environmental preferences. Compared to generalised species, a sample is more likely to capture the environmental conditions associated with a specialised vector occupying a smaller environmental domain, even at smaller sample sizes. This would improve predictive accuracy (32, 44). The clustered distribution of a vector due to specific environmental preferences should not to be confused with geographical clusters resulting from oversampling an area (18). Furthermore, Liu et al. (46) used a resolution of 100 m and model performance can also improve due to increased spatial accuracy, precise locations and greater delineation of habitats compared to our 1 km resolution (34, 36, 70).

Hendrickx et al. (18) used historical data for parasitic eggs in a host, as a proxy for VBD risk. They reported a minimum sample size of 1,516 (758 presence and absences each in the training subset), below which model performance rapidly deteriorated (18). The objectives to identify a minimum sample size differ from this study and thus, estimates for an optimum sample size would likely increase from 1,516, given that our findings suggested there was notable deterioration in model performance at a sample size of 100. Since their historical dataset was susceptible to geographical clustering and bias towards symptomatic cases (18), higher estimates are to be expected when predicting disease distribution in a host compared to the distribution of the virtual vector.

Considering the wider literature for RF models, our findings are more aligned with the estimates of 400–900 by Shiroyama et al. (45) and 592 by Tessarolo et al. (44). Both consider the number of presence and absences in the training subset. For balanced ratios, our estimates equate to 525–700 presence and absences in the training subset. However, both studies have differing methodologies. Tessarolo et al. (44) used historical datasets for 34 endemic terrestrial species and the estimates were reported for an ensemble of algorithms, which can perform better than a single algorithm (47, 71). Since Shiroyama et al. (45) predicted the distribution of a freshwater fish, the covariates and sampling methods are too disparate for reliable comparisons. Hanberry et al. (33) also reported an optimum sample size of 500 presences in the training subset but these estimates were obtained with a 4:1 ratio of presences and pseudo-absences (equivalent to 80:20), which was not considered in this study. Due to the scarcity of similar studies evaluating the effect of sample size on SDMs developed with RF, it was challenging to contextualise these findings with confidence due to differing model designs, data characteristics and species (Supplementary Tables 3, 4). While an extent of 10,000 km^2^ was a somewhat arbitrary cut-off, future research could consider accounting for these methodological differences in a meta-analysis. However, this may be a challenge without detailed, transparent methods reported in standardised protocols, such as ODMAP (48).

### 4.2 Poor model performance

Our findings indicate that sample sizes below 100 produced inaccurate models. Hanberry et al. (33) reported poor performance for models trained on fewer than 200 presence points, while Shiroyama et al. (45) noted a significant decrease in AUC below 100 presence and absence points in the training subset. At these sample sizes, training subsets are unlikely to be representative of the vector’s actual distribution. Broad geographic coverage and a representative range of environmental conditions in which the vector is present are key factors for accurate SDMs (44). The partition ratio may be a contributing factor since this determines the size of the training subset (which influences model accuracy) and the size of the test subset (which influences the risk of evaluation error). At partitioning, 70% of the dataset was assigned to the training subset and 30% to the test subset. This approach was taken to ensure there was sufficient data in the test subset to evaluate model performance at smaller sample sizes, reducing the risk of spurious results. This partitioning ratio has also been applied in other SDMs (72, 73). However, alternate approaches like k-fold partitioning may be more appropriate since this averages the results from several partitions and is less dependent on a single partition (68).

### 4.3 Performance by sample ratio

Between sample ratios, performance improved as the proportion of presence points increased. Other studies have reported similar findings (43), with balanced sample ratios producing the most accurate RF models (29, 47, 69). Poor model performance at small sample sizes was exacerbated for samples with a 20:80 ratio, as demonstrated by the large interquartile ranges which reflect varying performance between replicates (Supplementary Figure 2). RF can model complex, non-linear interactions between vector distribution and covariates, but becomes more susceptible to noise with less agreement between replicates at small sample sizes, particularly when the proportion of presence points decreases (29). Sample ratios with a greater proportion of absence points also performed better when evaluated by specificity, but this reflects bias towards the more prevalent class (presence or absence) on which the model was trained. Poor performance with unbalanced sample ratios is not restricted to RF, this is a well-known behaviour within machine learning, whereby models are sensitive to the majority class (presence or absence) (55).

Given the poor performance below a sample size of 100, particularly for unbalanced sample ratios, RF may not be suitable for modelling rare vectors. Maximum Entropy (MaxEnt) may be more appropriate since studies have reported reliable performance with this presence-background algorithm from sample sizes as low as three to 300 (32, 36–38, 40, 42). Conversely, several studies have also considered corrective methods for RF, such as down-sampling, to mitigate the effects of unbalanced sample ratios and improve model performance (55, 73). It is worth noting that the spatial presentation of model predictions (Figure 8; Supplementary Figures 4–9) showed indications of model overfitting with poor discrimination towards the southeast of the test site. While complex algorithms such as RF are prone to overfitting (44, 74), further research is warranted to determine if model accuracy at these smaller sample sizes and unbalanced ratios could be first improved by optimising model design.

### 4.4 Limitations

No model will ever be 100% accurate since SDMs are sensitive to data quality, assumptions and model design. SDMs are built on the principle that a vector has a specific environmental tolerance and therefore assumes that the abiotic covariates sufficiently describe its distribution in order to make predictions (27). During quality checks, an unexpected trend was observed: test sites 2, 5 and 9 frequently produced the least accurate models per sample size, while test sites 3, 6 and 7 produced the most accurate models per sample size. There was no apparent correlation with the ROA or geographical clustering of the vector’s actual or sampled distribution. To be ecologically appropriate, correlative SDMs depend on both the appropriate selection of covariates and samples which reflect the range of environmental conditions that the vector occupies (17, 44). For the former, if the selected covariates do not account for a species response to environmental gradients, model performance may deteriorate. The varied performance between test sites may be explained by different responses (abrupt versus smooth). Humidity and precipitation are two key climatic factors which can influence vector distribution (52), while land use is a key environmental variable (6, 52). These covariates were not included, which may merit further investigation to determine if their inclusion improves performance, but this was beyond the scope of this study. Our aim was to provide a general rule of thumb for the optimum sample size by grouping models across various test sites with differing habitat suitability and ROA. This approach ensures that any limitations in covariate selection were consistent across all sample sizes.

Climatic bias can reduce predictive accuracy if samples have restricted environmental variation and do not reflect the range of conditions that the vector inhabits. As such, Tessarolo et al. (44) argue that environmental coverage is more important than geographical coverage. Random sampling was among the least climatically biased designs when comparing seven sampling strategies (44). Although model performance varied little across the sampling strategie, biased sampling is expected to reduce accuracy to a greater extent for widespread, generalised species since these approaches are less likely to capture the range of environmental conditions that a vector inhabits, particularly at smaller sample sizes (44). While random sampling from a simulated vector with a fully known distribution should minimise this risk, future research should consider stratified random sampling along different environmental gradients. Random sampling minimises the risk of geographically clustered samples, but this is an idealised condition. For real vectors, not all locations are appropriate or accessible, and random sampling across large areas can be resource intensive. With a virtual vector, it is anticipated that our results would not differ significantly between stratified random sampling and random sampling. The influence of sampling strategy on model performance does constitute a separate research objective, but ensuring samples are both environmentally and geographically representative is an important consideration when applying our findings to real-world scenarios.

The optimum sample size was determined by thresholds for excellent model performance. While the five metrics are commonly used to evaluate SDMs, their thresholds can be subjective. Similar thresholds for PCC, sensitivity and specificity have been used in other studies (75, 76). However, Jiménez-Valverde et al. (28) caution that values of 0.6 can be obtained for Cohen’s Kappa by under- or overpredicting by 40% for a species with an ROA of 50%. Our thresholds for Cohen’s Kappa were based on those proposed by Landis and Koch (66), but McHugh (59) advocates for a more stringent criteria, suggesting that values between 0.60–0.79 actually represent moderate model performance, while values between 0.80–0.90 reflect strong model performance. This threshold for Cohen’s Kappa would impact our estimates since no model reached values between 0.80–0.90 for any sample size or ratio (Figure 7D). An alternative approach would involve calculating quartile deviations and determining the statistical significance of the differences between sample sizes (46). This approach would have also verified whether the point of diminishing returns observed between 1,500–2,000 and the model deterioration below a sample size of 100 truly reflected statistically marginal improvements in performance and a minimum sample size, respectively.

While emphasis was placed on thresholds for excellent model performance, reliable models may be developed with smaller sample sizes which meet the thresholds for moderate performance. Ultimately, the value of a model depends on the research objective and smaller sample sizes may still provide useful information if the intention is to explore areas with limited information on vector distribution or to prioritise field sampling of rare species (36, 42). To confirm the value of models at smaller sample sizes, future research should consider comparing the spatial distribution of incorrect predictions for vector distribution at smaller sample sizes to predictions developed with the optimum sample size. If inaccurate predictions are spread equally across the test site, rather than concentrated areas, models at smaller sample sizes would still provide useful information.

### 4.5 Potential impact

Model predictions act as a static risk map which can theoretically guide field sampling to locations with the highest probability of vector presence. In line with ECDC recommendations for SDMs, estimates for an optimum sample size provide a framework for strategic sampling, optimising the use of limited resources to both validate model predictions and improve the surveillance of vectors and their diseases (26). Our results can help researchers determine how many samples are needed for reliable models. Since abiotic variables are used to predict vector distribution, these results are most applicable to vectors that have strong associations with climatic and environmental factors. This may include established populations of both native and invasive vectors of significance to human health, animal health and food security, provided there are at least 375 presence and absence records each (for balanced ratios) in a test site.

Table 4 illustrates how the optimum sample size can guide field sampling, based on the expected probability of vector presence in a test site. However, caution is advised since confidence intervals have not been calculated for Table 4 which account for uncertainty and the sensitivity of sampling methods. The transferability of these findings to real vectors also warrants further investigation. Vector datasets are often collected without standardised sampling protocols and reliably sampling absence data in the field is challenging (16). If less sensitive sampling methods are employed, it may be necessary to collect a greater number of field samples to achieve the optimum number of presence and absences required for modelling. Our results offer general guidance for vector sampling programmes, but additional factors such as funding, resource availability, and time constraints must also be considered. Since historical datasets may contain only presence records, and are susceptible to opportunistic sampling targeting areas with reported disease or easily accessible locations, such as roads (36, 77), our optimum sample size estimates will likely increase for real vectors. While RF is fairly robust to convenience samples, Kessler et al. (78) caution against their use since model predictions for tick distributions were not sufficiently accurate for detailed decision making.

**Table 4:** Required number of field samples to obtain the optimum number of presences for a balanced sample ratio (50:50).

Sample size		Vector probability of presence								
Total	Presences	0.1	0.2	0.3	0.4	0.5	0.6	0.7	0.8	0.9
500	250	2,500	1,250	833	625	500	417	357	313	278
600	300	3,000	1,500	1,000	750	600	500	429	375	333
700	350	3,500	1,750	1,167	875	700	583	500	438	389
750	375	3,750	1,875	1,250	938	750	625	536	469	417
800	400	4,000	2,000	1,333	1,000	800	667	571	500	444
900	450	4,500	2,250	1,500	1,125	900	750	643	563	500
1,000	500	5,000	2,500	1,667	1,250	1,000	833	714	625	556
1,100	550	5,500	2,750	1,833	1,375	1,100	917	786	688	611
1,200	600	6,000	3,000	2,000	1,500	1,200	1,000	857	750	667
1,300	650	6,500	3,250	2,167	1,625	1,300	1,083	929	813	722

With an optimum sample size of 750–1,000 for a balanced sample ratio, 375–500 presences are required for excellent model performance (highlighted in yellow). Based on the expected probability of vector presence in the field, the number of samples which need to be collected will differ (calculated by dividing the number of required presence samples by the expected probability of vector presence).

SDMs ultimately depend on high-quality data and as outlined in the introduction, field sampling is costly and labour-intensive, particularly when collecting reliable absence data (16). While sufficient and comprehensive records on vector distribution are essential, model predictions can subsequently reduce costs by identifying strategic locations for future vector sampling programmes. As proposed by Lippi et al. (51), SDMs can be considered within a cyclical and iterative workflow. The increasing availability of vector presence and absence samples helps validate existing models, improves their predictive accuracy, and generates new risk maps for vector distribution. This, in turn, facilitates informed action by decision-makers and guides strategic sampling for new surveillance data.

## 5 Conclusion

We sought to evaluate the effect of sample size on model performance and to determine the optimum sample size for reliable Random Forest models which predict arthropod vector distribution. To the best of our knowledge, this is the first study which used a virtual vector and presence-absence data at this scale. A virtual vector overcomes limitations in data quality and confounding factors compared to field and historical datasets, facilitating evaluation with greater certainty. The optimum sample size estimates ranged from 750–1,000 for balanced samples and increased to 1,100–1,300 for samples with a 40:60 ratio of presence and absence points. Samples with a 20:80 ratio consistently produced unreliable models. Considering that the ROA and proportion of presence points in a sample have a large influence on model performance (44), accounting for these factors across 10 different test sites and three sample ratios was beneficial. Failure to consider the combined effects of factors may result in misleading conclusions. Since machine learning models vary slightly each time they are run (49), researchers should consider replicating models for stacked predictions, particularly when working with smaller sample sizes. While the optimum sample size may vary with different models, species and data characteristics, further research may first seek to refine or lower these estimates through optimised model design before determining how the optimum sample size differs for real vectors. Due to difficulties reliably sampling absence data in the field, it may be worthwhile investigating the effect of sample size on ratios with a greater proportion of presence points.

## Data Availability

The raw data supporting the conclusions of this article will be made available by the authors, without undue reservation.
