# Supplementary material for: Predicting vector distribution in Europe: at what sample size are species distribution models reliable?
Source: Front Vet Sci. 2025 May 29;12:1584864. doi: 10.3389/fvets.2025.1584864 (PMC12159067; doi:10.3389/fvets.2025.1584864)
Supplement: Supplementary file 1 [file Data_Sheet_1.docx]

Supplementary Material

# Supplementary Data

## R code

The R code supporting the conclusions of this article will be made available by the authors, without undue reservation.

## Rapid literature search

As part of a wider body of work for a MSc thesis, a rapid literature search was conducted to identify similar studies which evaluated the effect of sample size on species distribution models (SDMs). A rapid evidence synthesis approach was implemented, following streamlined methodologies with database searches, screening against an inclusion criteria and data extraction. Since the primary aim of this study was to evaluate the effect of sample size on Random Forest models, a systematic review was out of scope. Search terms were developed for three key terms: species distribution models, sample size and performance for three databases: Web of Science Core Collection, Scopus and PubMed. After conducting the search for title and abstract on the 03/05/2024, a filter for journal articles was applied to document type for all databases except PubMed, since relatively few results were retrieved. The search terms are provided in Supplementary Table 5-7. An inclusion criteria was used to identify primary peer-reviewed articles published in English with comparable methods (Supplementary Table 8). Comparable studies were considered to be SDMs developed with Random Forest at similar resolutions (equal to or less than 1km^2^) and extents (equal to or greater than 10,000km^2^). While species characteristics can influence performance, all terrestrial species were considered to ensure the search was not too restrictive. EndNote and Rayyan were used to deduplicate the studies and screening was conducted in Rayyan by one reviewer.

A total of 593 papers were retrieved. Following deduplication, title and abstract screening was conducted for 312 papers and 50 were assessed for eligibility on full text (Supplementary material Fig. 10). Overall, two studies met the inclusion criteria and evaluated the effect of sample size on fine resolution (≤1km^2^) and large-extent (≥10,000km^2^) Random Forest models with presence-absence data. An effort was made to identify comparable studies with a broad search strategy, but additional studies may be identified through citation searches and further database searches. It is important to note that screening and extraction was conducted by a single reviewer, which may introduce bias or limitations to this rapid search. While every effort was made to ensure rigor, the findings should be interpreted with caution since no critical appraisal was conducted.

# Supplementary Figures and Tables

## *Supplementary Figures*

**Supplementary Figure 1. Flowchart of methods.**

**
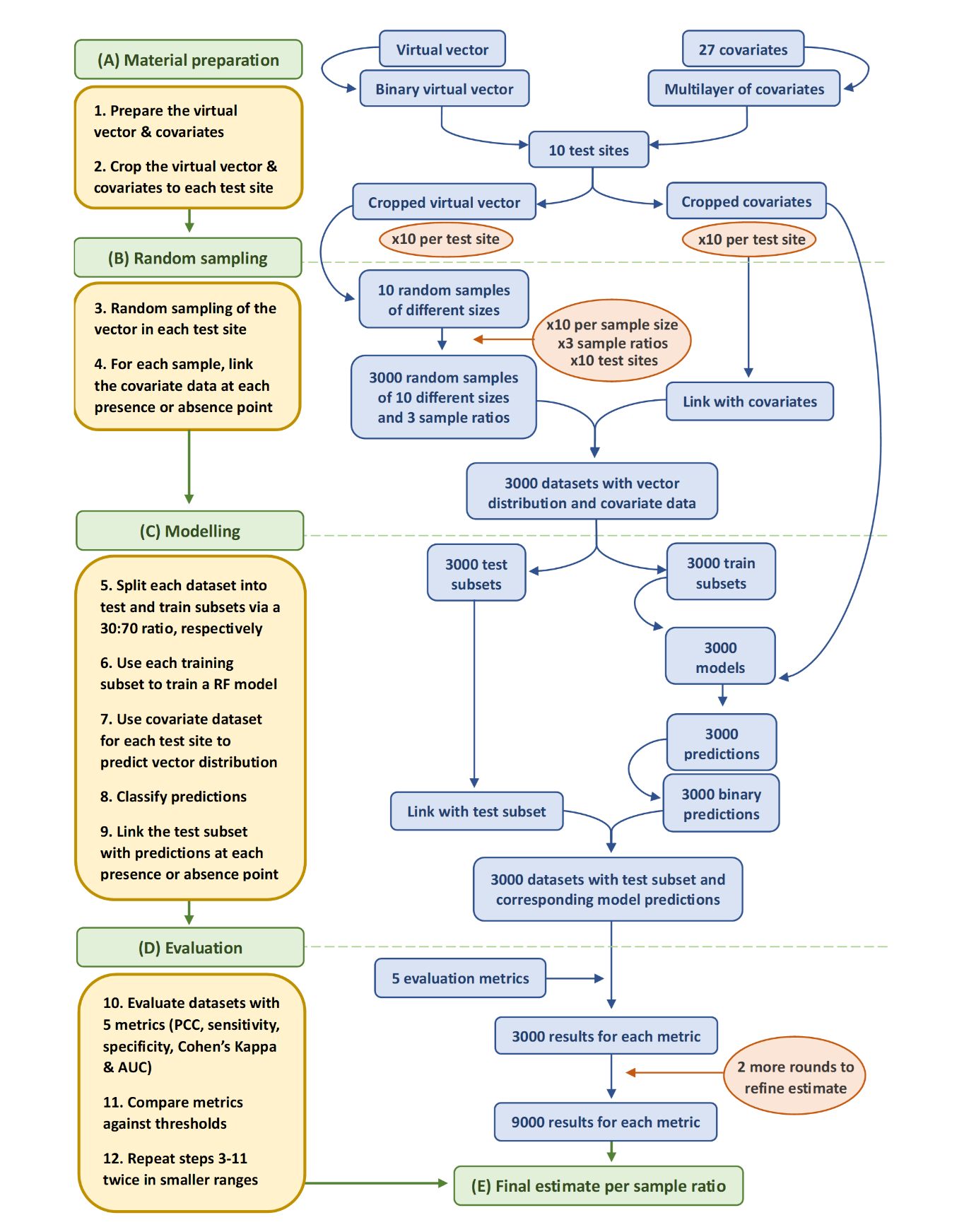
**

**Supplementary Figure 2. Round 1: Performance of 2999 models when evaluated by five metrics.** Each boxplot represents the means, medians and quartiles of 100 models, unless metrics could not be calculated. One model out of 3000 failed for a sample size of 10 with a 20:80 ratio. The models were grouped by 10 sample sizes (10, 30, 50, 80, 100, 250, 500, 1000, 2500 and 5000) and three sample ratios of presence and absence data (50:50, 20:80 and 40:60, respectively). The threshold bar represents the defined thresholds for excellent models (to three decimal points): 0.795 – 0.894 for **(A-C)** and **(E)** which present the PCC, sensitivity, specificity and AUC metrics, respectively, and 0.605 – 0.804 in **(D)** which presents Cohen’s Kappa.

**
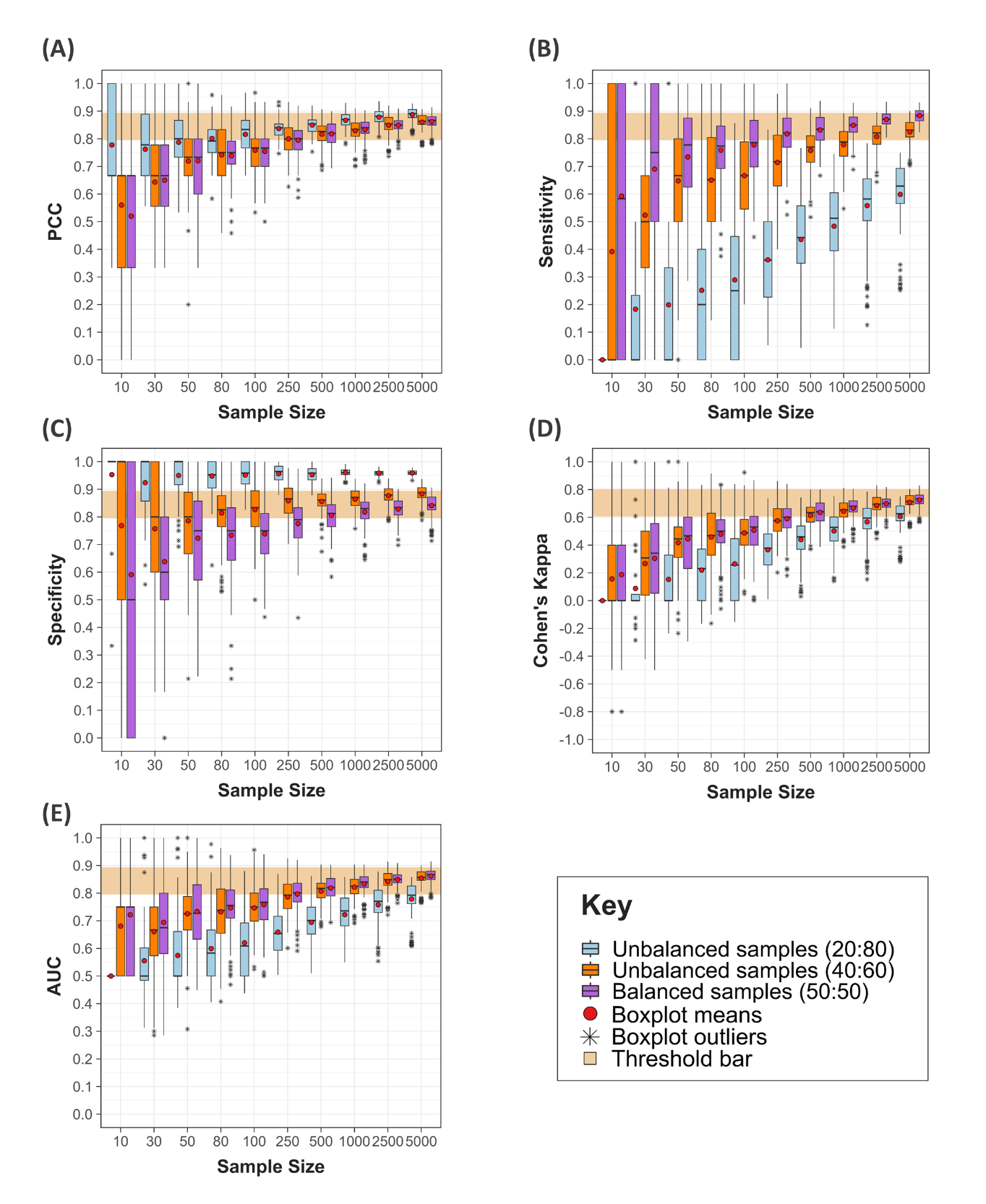
**

**Supplementary Figure 2 accompanying text:** Increasing sample size improved model performance across all sample ratios and evaluation metrics. The decrease in interquartile ranges above a sample size of 500 reflects less variation between replicates. Notably, the boxplots for sample sizes 10 and 30 display significant variation in their interquartile ranges and therefore, model performance, across all sample ratios and metrics. Supplementary figures 4-6 illustrate poor model discrimination between presence and absence at a sample size of 50, which improved with increasing sample size.

For unbalanced samples with a 20:80 ratio, no sample size reached the thresholds for sensitivity, Cohen’s Kappa, and AUC, but all exceeded the specificity threshold for excellent models. When evaluated by three out of five metrics, models with a 20:80 ratio were unreliable. Therefore, a narrower range of sample sizes was identified from models developed with 50:50 and 40:60 ratios. For unbalanced samples with a 40:60 ratio, the sample size which first met thresholds for excellent performance was 250, when evaluated by specificity (first quartile = 0.814, mean = 0.858) compared to a sample size of 5000, when evaluated by sensitivity (first quartile = 0.807, mean = 0.825). For balanced samples, the smallest sample size was 500, when evaluated by sensitivity (first quartile = 0.795, mean = 0.832) and largest was 2500, when evaluated by specificity (first quartile = 0.807, mean = 0.829). There are indications of a plateau above a sample size of 2500 across most metrics, suggesting there may be a point of diminishing returns. As such, the optimum sample size may fall between the range of 250 – 2500. This was increased to 150 – 3000 to account for a margin of error.

**Supplementary Figure 3. Round 2: Performance of 3000 models when evaluated by five metrics.** Each boxplot represents the means, medians and quartiles of 100 models which are grouped by 10 sample sizes (150, 250, 350, 500, 750, 1000, 1500, 2000, 2500 and 3000) and three sample ratios of presence and absence data (50:50, 20:80 and 40:60, respectively). The threshold bar represents the defined thresholds for excellent models (to three decimal points): 0.795 – 0.894 for **(A-C)** and **(E)** which present the PCC, sensitivity, specificity and AUC metrics, respectively, and 0.605 – 0.804 in **(D)** which presents Cohen’s Kappa.

**
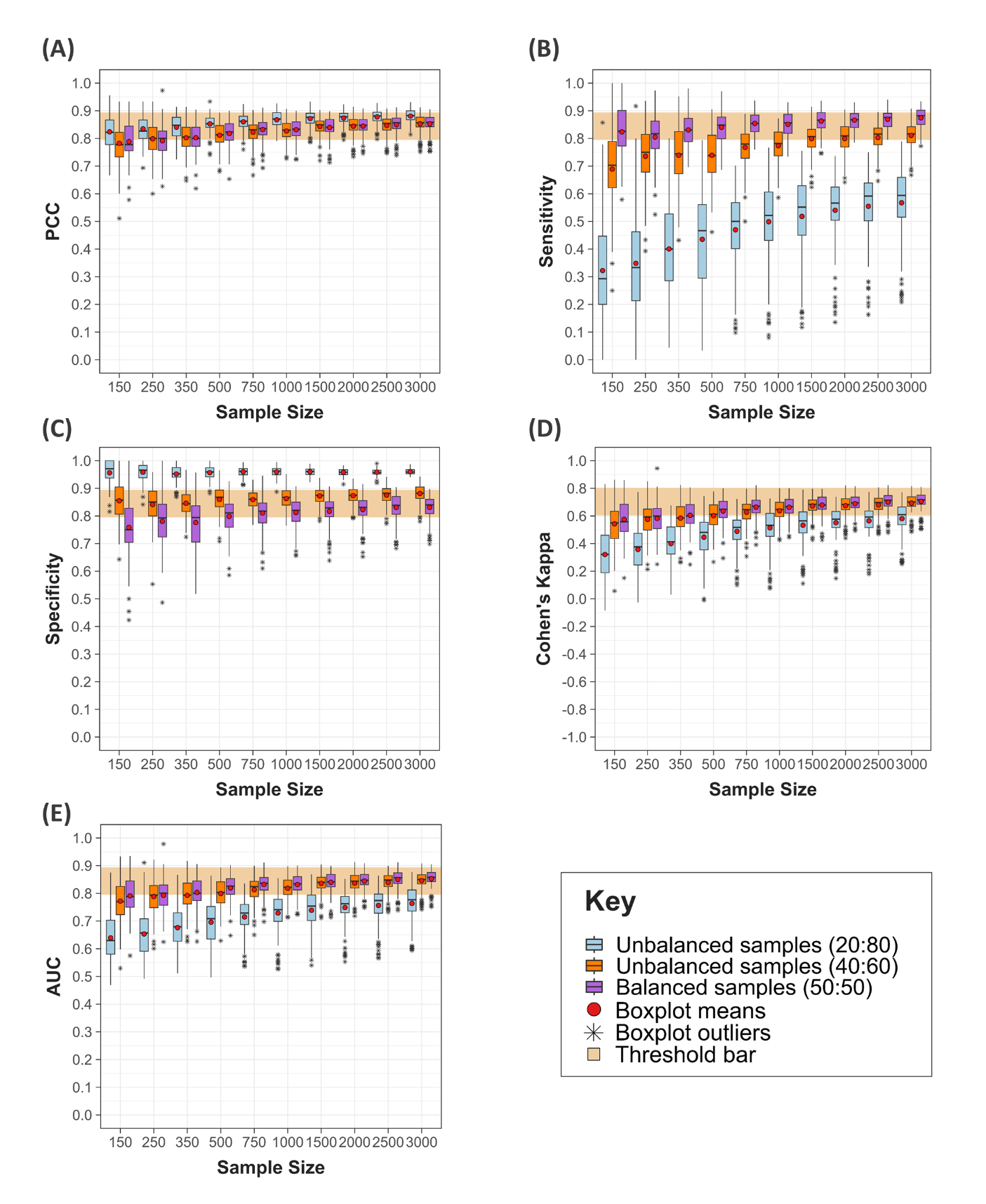
**

**Supplementary Figure 3 accompanying text:** Increasing sample size continued to improve model performance across all sample ratios and evaluation metrics. Like supplementary Figure 2, no sample size with a 20:80 ratio met the thresholds for sensitivity, Cohen’s Kappa and AUC. For unbalanced ratios with a 40:60 ratio, no sample size reached the sensitivity threshold, which is to be expected since a sample size of 5000 was the first to reach the threshold in the first round. The sample size with a 40:60 ratio which first met thresholds for excellent performance was 150, when evaluated by specificity (first quartile = 0.806, mean = 0.855) compared to 1500, when evaluated by Cohen’s Kappa (first quartile = 0.642, mean = 0.672). Compared to balanced ratios, the smallest sample size was 500 for both sensitivity (first quartile = 0.799, mean = 0.840) and AUC (first quartile = 0.796, mean = 0.819) and the largest was 1500, when evaluated by specificity (first quartile = 0.797, mean = 0.817).

However, important trends are missed when solely focusing on the first sample size to reach a threshold for excellent models. For a sample size of 250 and a 40:60 ratio, the first quartile dips to the threshold boundary for specificity (first quartile = 0.800, mean = 0.841), suggesting that models may be more reliable from sample sizes of 350 and above, instead of 150. When comparing boxplot position across all metrics, there were marginal improvements from sample sizes of 1500, which may suggest a point of diminishing returns. While samples with a 40:60 ratio first reached the threshold for Cohen’s Kappa at 1500, the first quartile for a sample size of 1000 was just below the threshold in the first round (first quartile = 0.603, mean = 0.644) and second round (first quartile = 0.596, mean = 0.638). Therefore, optimum performance may lie between 1000 - 1500 (supplementary Figs. 2-3). When accounting for poor performance with a 20:80 ratio and with less emphasis on specificity, the remaining metrics suggest the optimum sample size may lie between the range of 500 – 1000. To account for a degree of error, the range was extended to 400 – 1300.

**Supplementary Figure 4. Round 1: Spatial comparison of the virtual vector’s distribution against model predictions developed with balanced sample ratios (50:50) in test site 3.** The known distribution of the virtual vector **(A)** was compared to one model’s predictions for vector distribution when developed with a 50:50 sample ratio and **(B)** a sample size of 50, **(C)** a sample size of 500 and **(D)** a sample size of 5000. Each map represents increasing probabilities of presence per raster cell, categorised into five classes using equal intervals.


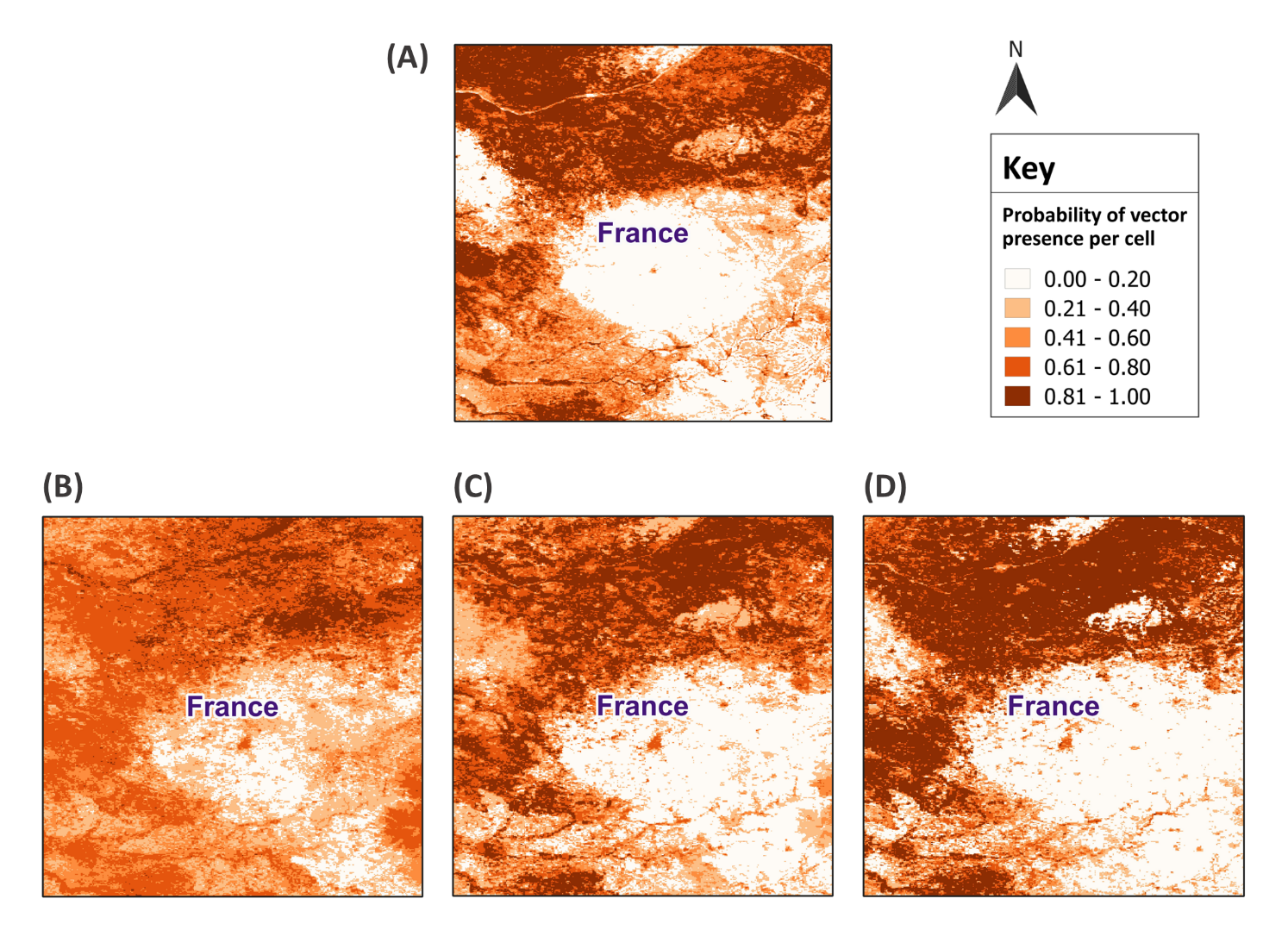


**Supplementary Figure 5. Round 1: Spatial comparison of the virtual vector’s distribution against model predictions developed with unbalanced sample ratios (20:80) in test site 3.** The known distribution of the virtual vector **(A)** was compared to one model’s predictions for vector distribution when developed with a 20:80 sample ratio and **(B)** a sample size of 50, **(C)** a sample size of 500 and **(D)** a sample size of 5000. Each map represents increasing probabilities of presence per raster cell, categorised into five classes using equal intervals.


**
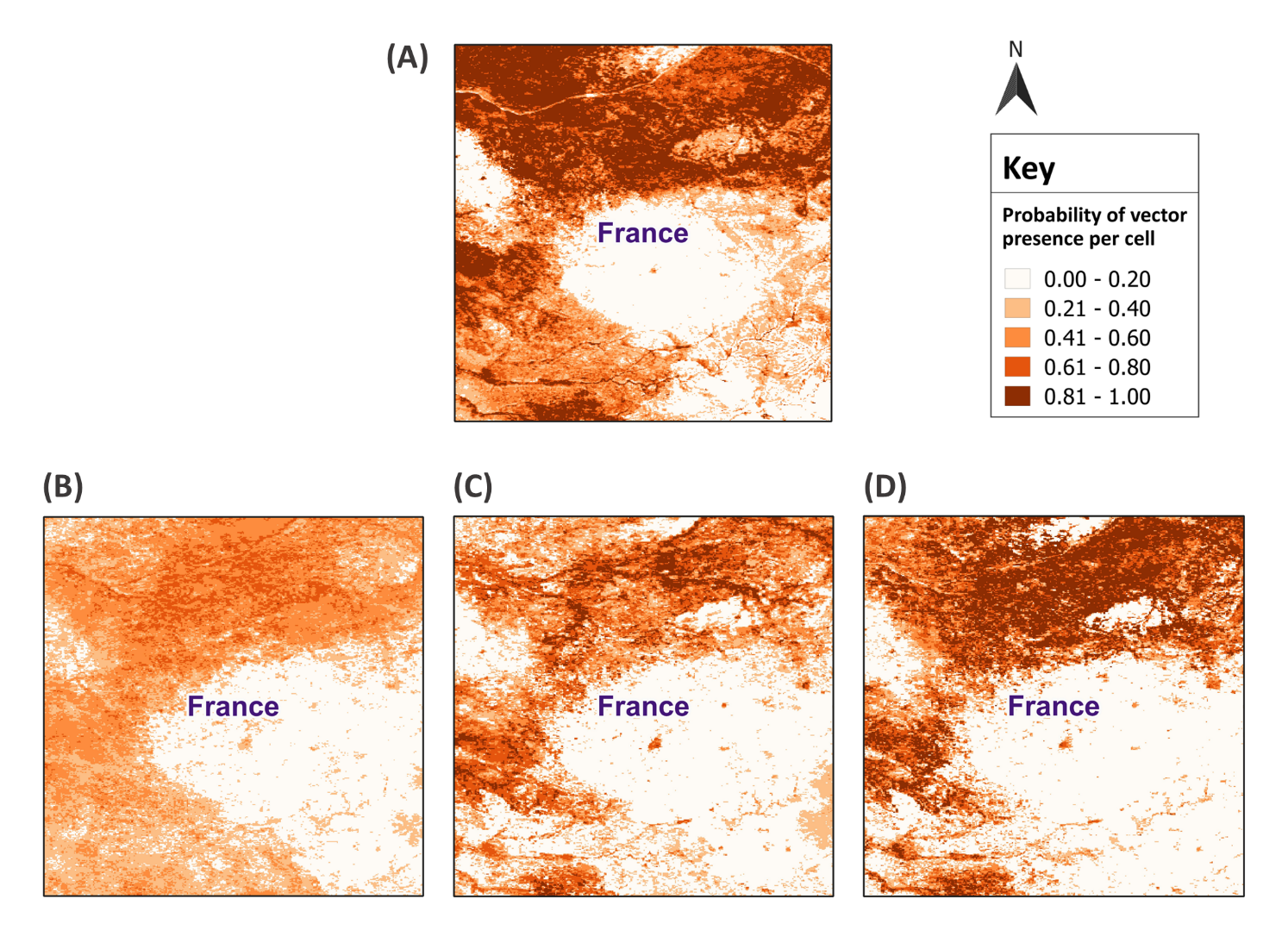
**

**Supplementary Figure 6. Round 1: Spatial comparison of the virtual vector’s distribution against model predictions developed with unbalanced sample ratios (40:60) in test site 3.** The known distribution of the virtual vector **(A)** was compared to one model’s predictions for vector distribution when developed with a 40:60 sample ratio and **(B)** a sample size of 50, **(C)** a sample size of 500 and **(D)** a sample size of 5000. Each map represents increasing probabilities of presence per raster cell, categorised into five classes using equal intervals.

**
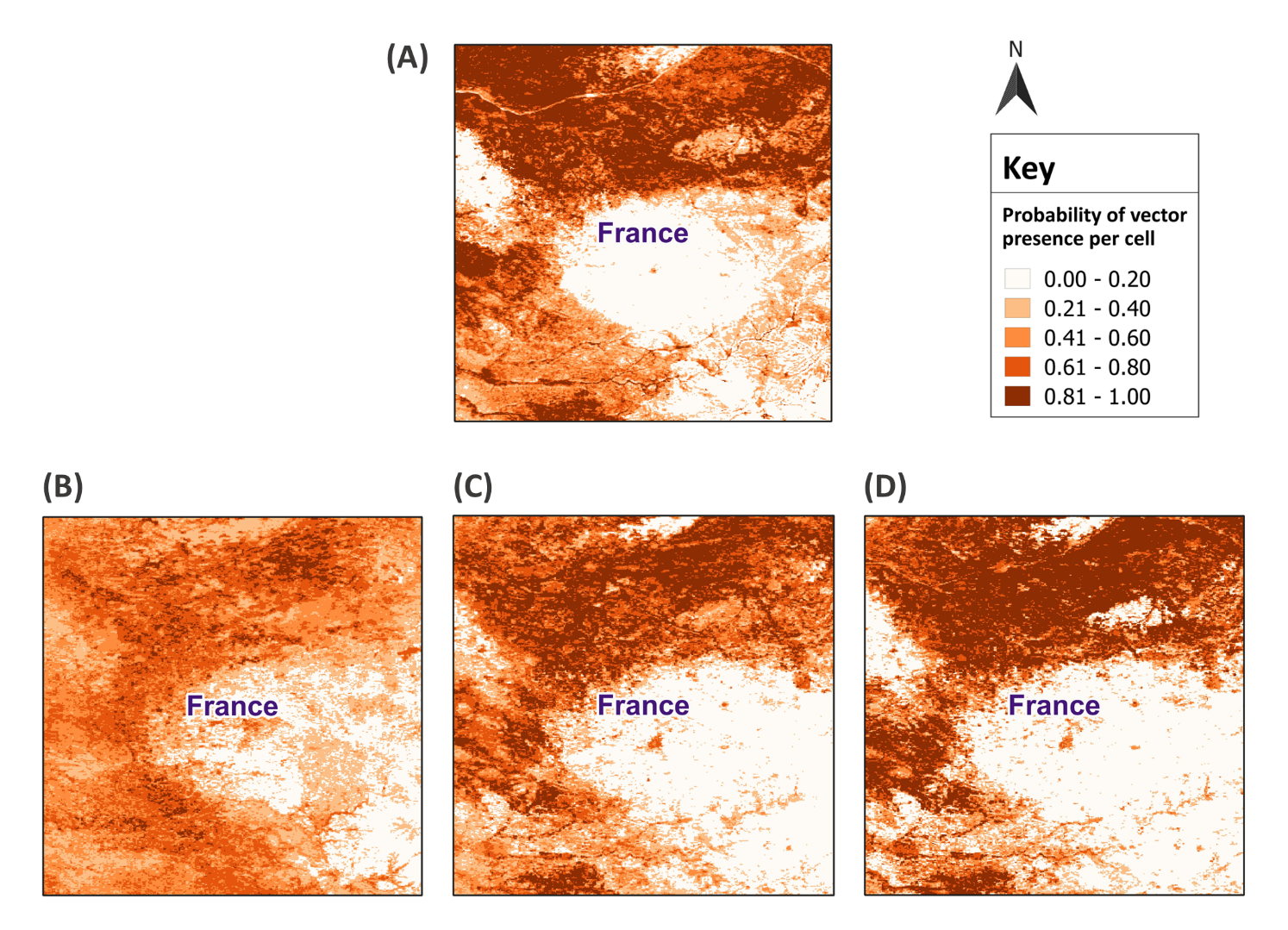
**

**Supplementary Figure 7. Round 2: Spatial comparison of the virtual vector’s distribution against model predictions developed with balanced sample ratios (50:50) in test site 3.** The known distribution of the virtual vector **(A)** was compared to one model’s predictions for vector distribution when developed with a 50:50 sample ratio and **(B)** a sample size of 250, **(C)** a sample size of 1000 and **(D)** a sample size of 2500. Each map represents increasing probabilities of presence per raster cell, categorised into five classes using equal intervals.

**
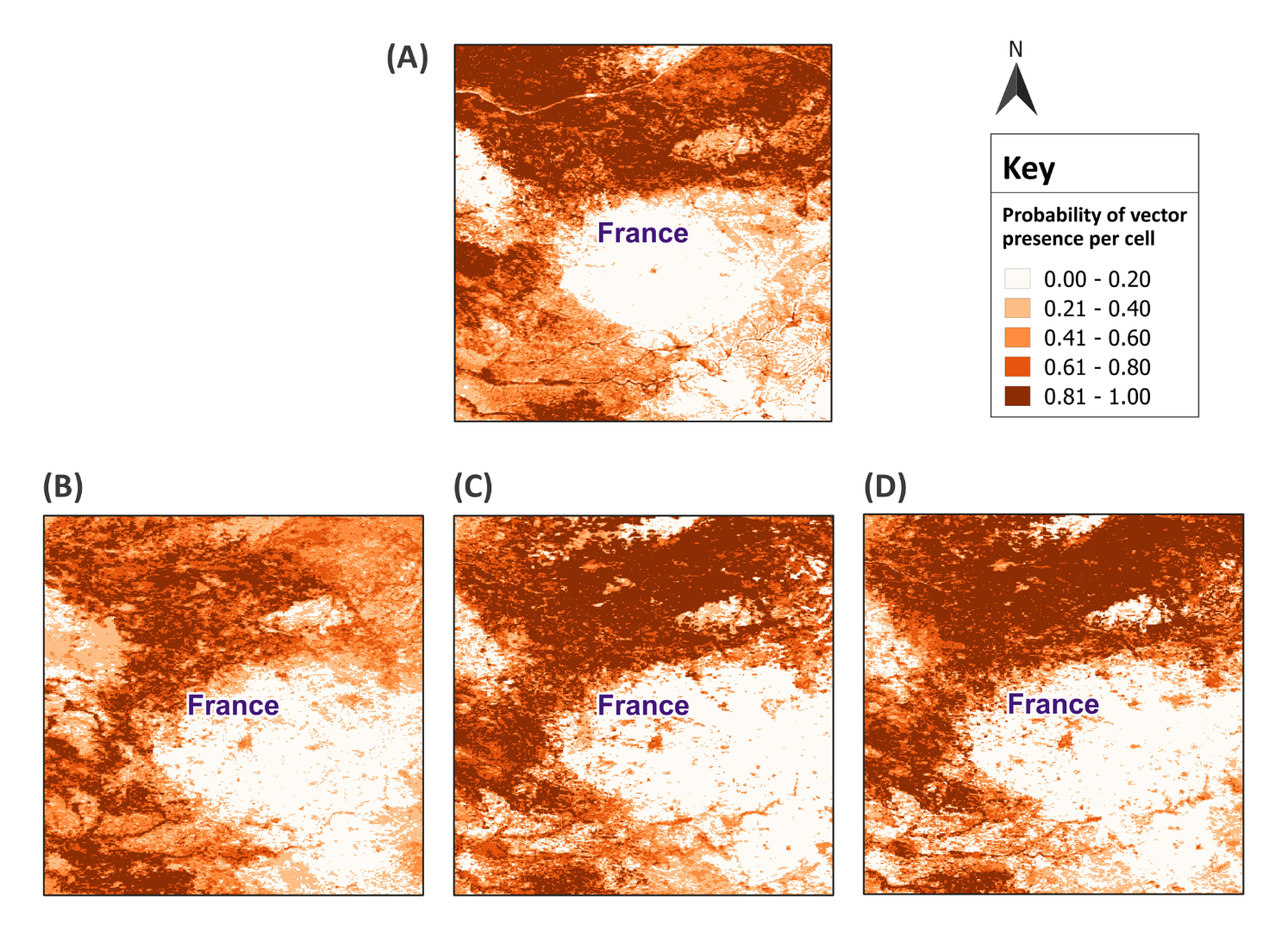
**

**Supplementary Figure 8. Round 2: Spatial comparison of the virtual vector’s distribution against model predictions developed with unbalanced sample ratios (20:80) in test site 3.** The known distribution of the virtual vector **(A)** was compared to one model’s predictions for vector distribution when developed with a 20:80 sample ratio and **(B)** a sample size of 250, **(C)** a sample size of 1000 and **(D)** a sample size of 2500. Each map represents increasing probabilities of presence per raster cell, categorised into five classes using equal intervals.

**
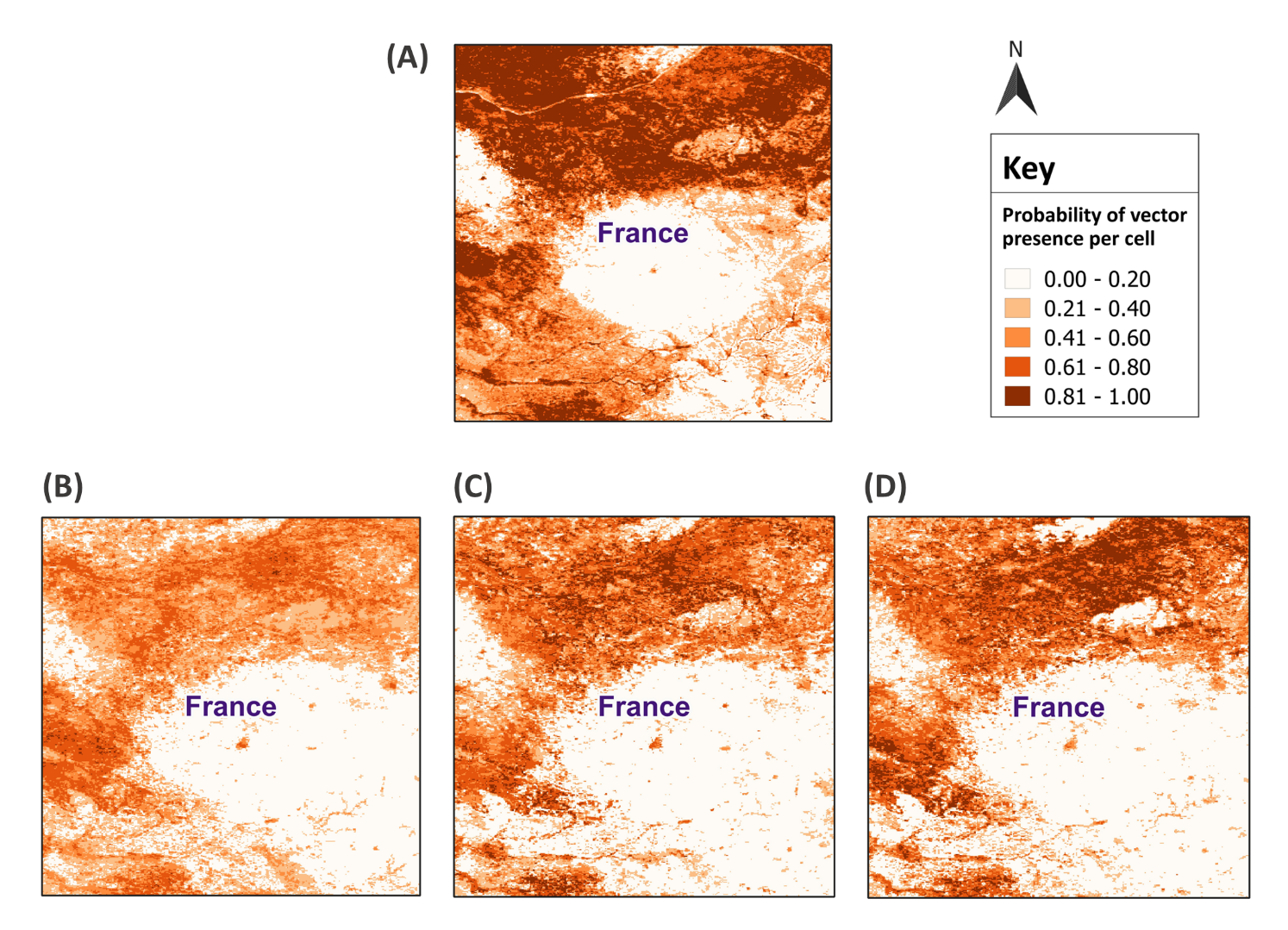
**

**Supplementary Figure 9. Round 2: Spatial comparison of the virtual vector’s distribution against model predictions developed with unbalanced sample ratios (40:60) in test site 3.** The known distribution of the virtual vector **(A)** was compared to one model’s predictions for vector distribution when developed with a 40:60 sample ratio and **(B)** a sample size of 250, **(C)** a sample size of 1000 and **(D)** a sample size of 2500. Each map represents increasing probabilities of presence per raster cell, categorised into five classes using equal intervals.


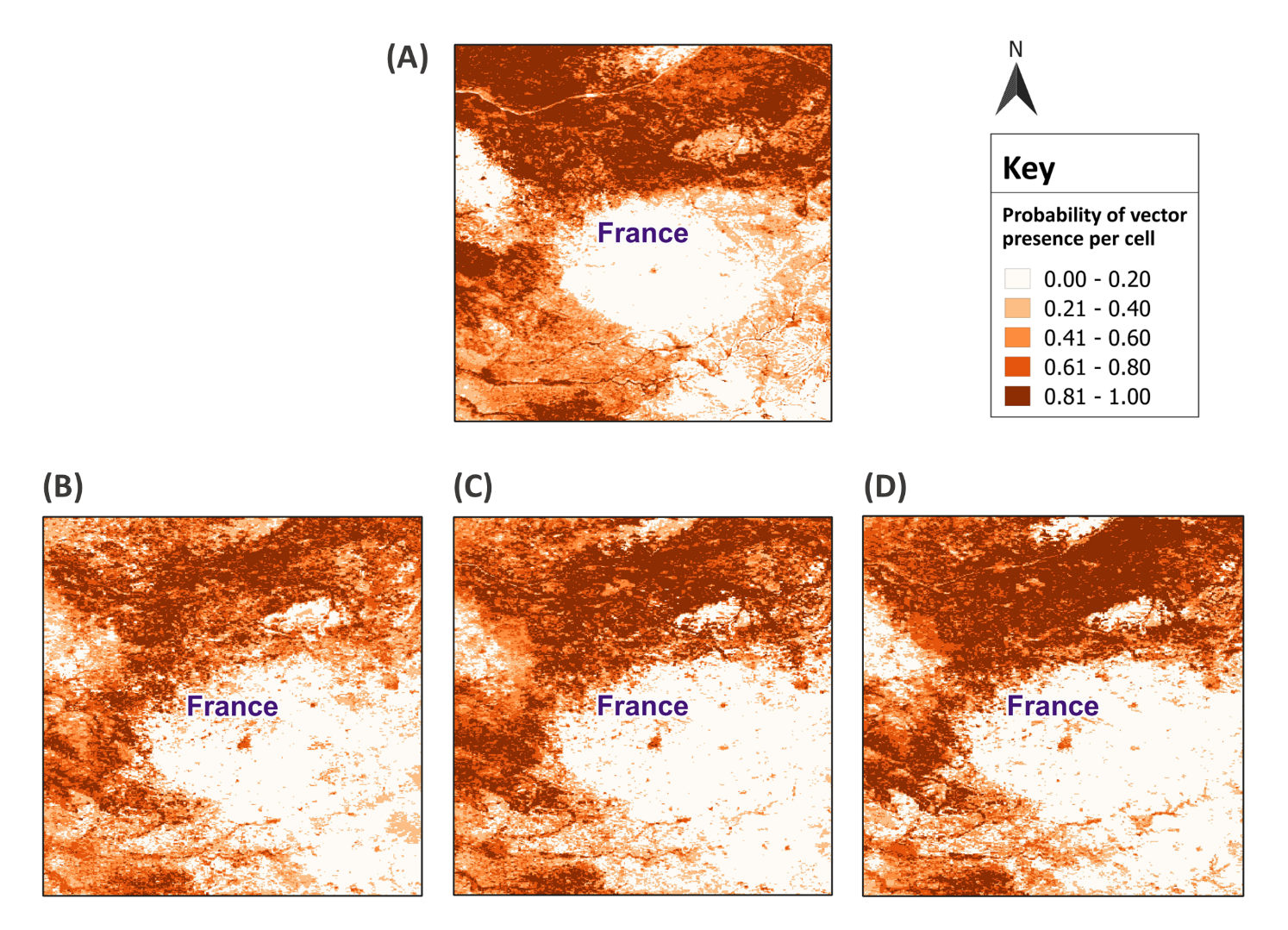


**Supplementary Figure 10.** **PRISMA 2020 diagram.** Adapted from the PRISMA diagram for new systematic reviews, which only include searches of databases and registers (1).


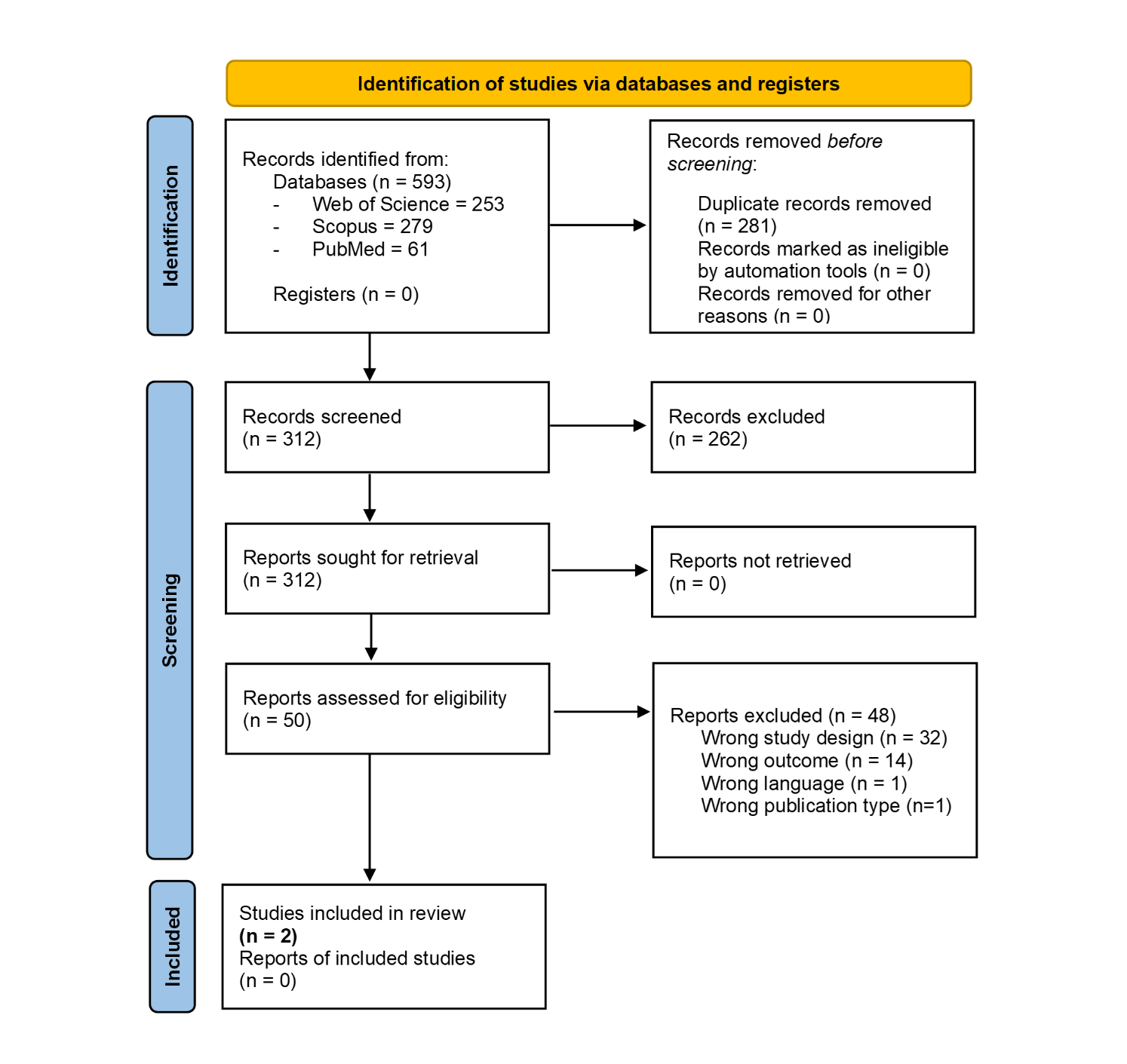


## *Supplementary Tables*

**Supplementary Table 1**. **Full ODMAP reporting protocol for SDMs** (48).

| ODMAP Criteria | Content |
| --- | --- |
| Overview Model objective | ***Objective*:** Mapping and interpolation  ***Target output:*** Binary maps of vector presence and absence in 10 test sites across Europe |
| Focal Taxon | Simulated virtual vector |
| Location | 10 test sites across mainland Europe |
| Scale of analysis | ***Spatial extent:*** Virtual vector has a spatial extent of -13.0°W, 43.0°E, 29.0°N, 72.0°N which was cropped to the following extents:  - Test site 1: -8.6°W, -5.3°W, 37.4°N, 40.7°N  - Test site 2: -3.8°W, -0.5°W, 39.7°N, 43.0°N  - Test site 3: -0.5°W, 2.8°E, 44.5°N, 47.8°N  - Test site 4: 4.1°E, 7.4°E, 47.8°N, 51.2°N  - Test site 5: 8.0°E, 11.4°E, 49.8°N, 53.2°N  - Test site 6: 11.9°E, 15.2°E, 49.8°N, 53.2°N  - Test site 7: 15.6°E, 18.9°E, 46.5°N, 49.9°N  - Test site 8: 19.7°E, 23.0°E, 45.2°N, 48.6°N  - Test site 9: 19.7°E, 23.0°E, 41.1°N, 44.4°N  - Test site 10: 23.8°E, 27.1°E, 41.1°N, 44.4°N  ***Spatial resolution:*** 0.0083° x 0.0083° (approximately 1 km x 1km at the equator) ***Temporal extent:*** 2001 – 2021  ***Boundary:*** 10 square test sites with an area of 11.111°^2^ (approximately 136,900 km^2^ at the equator) |
| Biodiversity data | ***Observation type:*** 100% known distribution (probability of presence per raster cell) ***Response data type:*** Presence/absence |
| Predictors | ***Predictor types:*** Climatic and vegetation |
| Hypotheses | ***Hypotheses:*** A virtual vector with a 100% known distribution can be used to evaluate the effect of sample size on the accuracy of SDMs developed with the Random Forest algorithm. Since the virtual vector was built to respond to climatic conditions, models will form associations between the covariates and vector presence / absence and predict distribution where sampling did not occur in each test site. |
| Assumptions | ***Model assumptions:*** - The use of a virtual vector with a 100% known distribution for presence and absence facilitates true random sampling and removes spatial bias.  - The chosen covariates sufficiently describe the relationship between abiotic factors and vector distribution in each of the 10 test sites. - Replication will sufficiently minimise the risk of spurious results, particularly for small sample sizes. - Grouping evaluation metrics by sample size and sample ratio will account for differing habitat suitability and the vector’s relative occurrence area in the 10 test sites.  - Any limitations with data quality or methodology will be the same across all SDMs created. |
| Algorithms | ***Modelling techniques:*** Regression Random Forest ***Model complexity:*** 500 trees with an optimum number of variables at each split (chosen by tuning mtry with the tuneRF function and default settings). ***Model averaging:*** Not applicable |
| Workflow | ***Model workflow:*** After material preparation (dichotomising the virtual vector to binary values for presence and absence using a threshold of 0.5 and cropping the virtual vector and covariates to each test site), 9000 random samples were created with 24 different sample sizes and three sample ratios. Each presence or absence point was linked to the covariates before each sample was partitioned into test and training subsamples using a 30:70 ratio, respectively. One model failed so 8999 Random Forest models were fit using the training subsamples and the covariate dataset to make predictions for vector distribution across each respective test site, where sampling did not occur. Predictions were evaluated using the test subsample and five metrics to identify the optimum sample size at which SDMs made accurate predictions. |
| Software | ***Software:*** R version 4.3.1. For packages, refer to Supplementary Table 2. ***Code availability:*** The R code supporting the conclusions of this article will be made available by the authors, without undue reservation. |
| Data |  |
| Biodiversity data | ***Taxon names:*** Not applicable since a virtual vector was used. ***Taxonomic reference system:*** Not applicable. ***Ecological level:*** Species.  ***Data sources:*** Created using the probability approach for SDMvspecies algorithm in R. ***Sampling design:*** Random sampling without replacement within each of the 10 test sites. ***Sample size:*** 9000 samples with 24 different sample sizes, defined as the total number of presence and absence points in the overall dataset, before partitioning (10, 30, 50, 80, 100, 150, 250, 350, 400, 500, 600, 700, 750, 800, 900, 1000, 1100, 1200, 1300, 1500, 2000, 2500, 3000, 5000). Three sample ratios were used, with 3000 samples each, which determines the proportion of presence and absence points in each sample (balanced datasets with a 50:50 ratio, and unbalanced datasets with a 20:80 and 40:60 ratio of presence and absence points, respectively). ***Clipping:*** Data was clipped to 10 test sites of varying extents (long/lat). Refer to spatial extent. ***Absence data:*** Since the virtual vector has a 100% known distribution data, the dataset contains true absences which have an equal probability of sampling as the presence points. ***Background data:*** Not applicable. ***Errors and biases:*** Each of the 10 test sites have varying habitat suitability and spatial distribution of the vector which may result in the geographical clustering of presence and absence points during random sampling, but performance was evaluated by grouping sample size across the test sites to account for this. |
| Data partitioning | ***Training data:*** 70% of each of the 9000 samples were randomly split into training subsamples without replacement. ***Validation data:*** 30% of each of the 9000 samples were randomly split into test subsamples, without replacement. These were withheld from model fitting and used to validate model performance. The proportion was decided to ensure there were sufficient observations to train the model, while retaining enough data to evaluate predictive accuracy at smaller sample sizes. |
| Predictor variables | ***Predictor variables:*** Covariates which were temporal Fourier transformed. ***Data sources:*** MODIS satellite imagery ***Spatial extent:*** -73.26°W, 69.07°E, 18.97°N, 83.63°N ***Spatial resolution:*** 0.0083° x 0.0083° (approximately 1 km x 1km at the equator) ***Coordinate reference system:*** EPSG:4326 – WGS 84 ***Temporal extent:*** 2001 - 2021 ***Data processing:*** Each covariate was cropped to each of the test sites’ extents. |
| Transfer data | ***Data sources:*** Not applicable  ***Spatial extent:*** Not applicable ***Spatial resolution:*** Not applicable  ***Temporal extent:*** Not applicable  ***Quantification of novelty:*** Not applicable |
| Model |  |
| Variable pre-selection | ***Variable pre-selection:*** The choice of covariates was based on expert advice and literature support. One set of covariates was chosen to minimise the risk of overfitting models. |
| Multicollinearity | ***Multicollinearity:*** The covariates were designed to account for collinearity and are independent, so multicollinearity was not addressed. |
| Model settings | ***Regression Random Forest:*** Default settings were used, except for the number of trees and number of variables at each node (randomForest package version 4.7-1.1): - Number of trees (500)  - Number of variables (Optimum number identified by tuning mtry with the tuneRF function and default settings) ***Model settings (extrapolation):*** Not applicable |
| Model estimates | ***Coefficients:*** Not applicable ***Parameter uncertainty:*** Not applicable ***Variable importance:*** Calculated but not assessed |
| Analysis and correction of non-independence | ***Spatial autocorrelation:*** Not conducted ***Temporal autocorrelation:*** Not conducted ***Nested data:*** Not applicable |
| Threshold selection | ***Threshold selection:*** Continuous predictions were converted into binary values using a threshold of 0.5 (probability of presence greater than 0.5 was assigned a value of 1 for presence and a probability of presence equal to, or less than, 0.5 was assigned a value of 0 for absence). This was chosen to evaluate model predictions against the known distribution of the virtual vector using five threshold dependent and independent metrics. |
| Assessment |  |
| Performance statistics | ***Performance on training data:*** Not applicable ***Performance on validation data:*** PCC, Sensitivity, Specificity, Cohen’s Kappa and AUC ***Performance on test data:*** Not applicable |
| Plausibility checks | ***Response shapes:*** Not conducted since the virtual vector provides a 100% known distribution and therefore, ecological plausibility checks of the models were not required. ***Expert judgement:*** Spatial predictions were visually compared against the 100% known distribution, but evaluations were predominantly based on the performance metrics. |
| Prediction |  |
| Prediction output | ***Prediction unit:*** Not applicable |
| Uncertainty quantification | ***Algorithmic uncertainty:*** Not applicable ***Input data uncertainty:*** Not applicable ***Parameter uncertainty:*** Not applicable ***Scenario uncertainty:*** Not applicable ***Novel environments:*** Not applicable |

**Supplementary Table 2.** **Packages and their key functions in R.**

| Package name | Version | Main functions | Source |
| --- | --- | --- | --- |
| base | 4.3.1 | ifelse, for, set.seed, sample, factor, table, rbind, data.frame, c, nrow, ncol, length, round, sum, diag, rowSums, colSums, is.na and mean | (2) |
| terra | 1.7-39 | as.polygons, crop, extract, spatSample, as.data.frame, which.min and predict | (3) |
| randomForest | 4.7-1.1 | tuneRF, randomForest and varImpPlot | (4) |
| PresenceAbsence | 1.1.11 | pcc | (5) |
| caret | 6.0-94 | sensitivity and specificity | (6) |
| vcd | 1.4-11 | Kappa | (7) |
| pROC | 1.18.4 | roc and auc | (8) |
| dplyr | 1.1.2 | mutate, select, group_by and summarize | (9) |
| RColorBrewer | 1.1-3 | display.brewer.all, display.brewer.pal and brewer.pal | (10) |
| ggplot2 | 3.4.2 | ggplot | (11) |

Supplementary Table 3. Methodological differences between publications which evaluated the effect of sample size on Random Forest models. All were compared to the methods implemented in this study. If information was not reported or not applicable, this was denoted by N/A.

*^1^ Publication was excluded due to incomparable methods
*^2^ Compared to the thresholds for excellent performance, as defined in this study
*^3^ Estimates for the optimum sample size did not use the same sample ratio, but the author’s earlier objective to identify the optimum sample ratio did use at least one of the same ratios

| Model design (compared to our study) | Author & Year | | | | | | | | |
| --- | --- | --- | --- | --- | --- | --- | --- | --- | --- |
|  | **Hendrickx *et al.* (12)** | **Liu *et al.* (13)** | **Liu *et al.* (14)** ***^1^** | **Grimmett *et al.* (15)** ***^1^** | **Hanberry *et al.* (16)** ***^1^** | **Santini *et al.* (17)** ***^1^** | **Shiroyama *et al.* (18)** ***^1^** | **Soultan and Safi (19)** ***^1^** | **Tessarolo *et al.* (20)** ***^1^** |
| Virtual vector | _🗶_ | _🗶_ | **_🗸_** | **_🗸_** | _🗶_ | **_🗸_** | _🗶_ | **_🗸_** | _🗶_ |
| Resolution ≤ 1km^2^ | **_🗸_** | **_🗸_** | **_🗸_** | **_🗸_** | _🗶_ | _🗶_ | **N/A** | _🗶_ | _🗶_ |
| Extent ≥ 10,000km^2^ | **_🗸_** | **_🗸_** | **_🗸_** | **_🗸_** | **_🗸_** | **_🗸_** | **_🗸_** | **_🗸_** | **_🗸_** |
| Presence-absence data | **_🗸_** | **_🗸_** | _🗶_ | _🗶_ | _🗶_ | _🗶_ | **_🗸_** | _🗶_ | **_🗸_** |
| Sample size: number of presence and absences before partitioning | _🗶_ | _🗶_ | _🗶_ | _🗶_ | _🗶_ | _🗶_ | _🗶_ | _🗶_ | _🗶_ |
| Same sample ratios (at least one) | **_🗸_** | _🗶_*^3^ | _🗶_ | **_🗸_** | _🗶_ | _🗶_ | **N/A** | **N/A** | **N/A** |
| Binary classification threshold of 0.5 | **N/A** | **_🗸_** | _🗶_ | _🗶_ | _🗶_ | _🗶_ | **N/A** | _🗶_ | _🗶_ |
| Same evaluation metrics (at least one) | **_🗸_** | **_🗸_** | **_🗸_** | **_🗸_** | **_🗸_** | **_🗸_** | **_🗸_** | **_🗸_** | **_🗸_** |
| Sample size reached excellent threshold*^2^ | _🗶_ | **_🗸_** | **N/A** | **N/A** | **N/A** | **N/A** | **_🗸_** | **_🗸_** | **N/A** |
| ODMAP protocol | **_🗸_** | _🗶_ | _🗶_ | _🗶_ | _🗶_ | _🗶_ | _🗶_ | _🗶_ | _🗶_ |

**Supplementary Table 4**. **Summary of publications which evaluated the effect of sample size on Random Forest models.** If information was not reported or not applicable, this was denoted by N/A. This includes metrics which are presented in figures when the value of a metric at the minimum or optimum sample size cannot be deduced.

*^1^ Publication was excluded due to incomparable methods
*^2^ Caution: sample sizes were defined differently between studies

| Author & Year | Species & Region | Sample sizes *^2^ | Sample ratio | Outcome | Metrics for the minimum or optimum size |
| --- | --- | --- | --- | --- | --- |
| Hendrickx *et al.* (12) | Species: Trematode eggs (*Dicrocoelium dendriticum*) in sheep (the host)  Region: Italy | 10 sizes (258, 508, 758, 1008, 1258, 1508, 1758, 2008, 2258 and 2508)  Definition: presences in training subset | 50:50 presence: absence | Minimum: 1,516 presence and absences (758 each) | Sensitivity: 0.62 Specificity: 0.60 Kappa: 0.48 AUC: 0.67 |
| Liu *et al.* (13) | Species: Snail (*Oncomelania hupensis*)  Region: Anhui region, China | 100 sizes (increments of 120 between 120 – 12,000)  Definition: inferred presence and absences in training subset | 1:2 presence: absence | Optimum: 2,400 presence and absences | PCC: N/A Sensitivity: N/A Specificity: N/A Cohen’s Kappa: N/A AUC: 0.96 |
| Liu *et al.* (14) *^1^ | Species: 600 virtual species when evaluating the number of presences on individual model performance  Region: Victoria, Australia | Nine sizes reported when evaluating the number of presences on individual model accuracy (10, 20, 40, 80, 160, 320, 640, 1280 and 2560)  Definition: presences in the training subset | 1:2 presence: background / pseudo-absences &   5120 background points / pseudo-absences at all sizes | General trends reported | General trends reported but authors used sensitivity, specificity, AUC, point biserial correlation coefficient and true skill statistic |
| Grimmett *et al.* (15) *^1^ | Species: 10 virtual species  Region: NSW and Victoria, Australia | Five sizes (20, 40, 60, 80 and 100)  Definition: presences in training subset | 5 ratios of presence to background points with proportion of presences set at 0.005, 0.01, 0.05, 0.1 and 0.5 | General trends reported | General trends reported but authors used sensitivity, specificity, Cohen’s Kappa, AUC, Pearson’s correlation coefficient and Fleiss’ Kappa |
| Hanberry *et al.* (16) *^1^ | Species: 16 tree species  Region: Minnesota’s Laurentian Mixed Forest, USA | Eight sizes (30, 50, 100, 200, 500, 1000, 1250 and 2500)  Definition: inferred presences in training subset | 80:20 presence: pseudo-absence | Optimum (inferred): 500 presences | Weighted Kappa: N/A Pearson’s *r*: N/A Cronbach’s alpha: N/A Absolute agreement metric (ICC2): N/A Consistency metric (ICC3): N/A |
| Santini *et al.* (17) *^1^ | Species: 50 virtual species  Region: Global (random locations) | Seven sizes (10, 25, 50, 100, 250, 500 and 1,000)  Definition: presences in training subset | 3 ratios of presence to background points with proportion of presences set at 0.01, 0.1 and 1 | General trends reported but inferred an optimum size of 200 – 500 points (not specific to RF, across three algorithms) | General trends reported but authors used AUC and true skill statistic |
| Shiroyama *et al.* (18) *^1^ | Species: Freshwater fish (*Lepomis macrochirus*)  Region: Seven rivers, Kanto region, Japan | 14 sizes (50, 60, 70, 80, 90, 100, 200, 300, 400, 500, 600, 700, 800 and 900)  Definition: presence and absences in training subset | N/A   (15% of overall dataset contained presences) | Optimum (inferred): 400 – 900 presence and absences | AUC means at 400: N/A AUC means at 900: 0.85 |
| Soultan and Safi (19) *^1^ | Species: Four virtual species  Region: Africa | Six sizes (5, 10, 20, 50, 100 and 200)  Definition: presences in training subset | N/A | Minimum: 20 – 100 from specialist to generalist species | Specialist (high resolution) at sample size 20: AUC: 0.954 True skill statistic (TSS): 0.909 Overall Concordance Correlation Coefficient (OCCC): 0.454 *Schoener’s D* index: 0.229  Relaxed specialist (high resolution) at sample size 20: AUC: 0.732 TSS: 0.488 OCCC: 0.359 *Schoener’s D* index: 0.328  Restricted generalist (high resolution) at sample size 100:  AUC: 0.852 TSS: 0.626 OCCC: 0.502 *Schoener’s D* index: 0.56  Generalist (high resolution) at sample size 100: AUC: 0.765 TSS: 0.483 OCCC: 0.299 *Schoener’s D* index: 0.616 |
| Tessarolo *et al.* (20) *^1^ | Species: 34 terrestrial species  Region: Iberian Peninsula | Five sizes reported as % of 5,919 cells (equates to 59 (1%), 296 (5%), 592 (10%), 1184 (20%) & 1480 (25%))  Definition: presence and absences in training subset | N/A | Optimum (inferred): 592 presence and absences for an ensemble of eight algorithms (including RF) | PCC: N/A Sensitivity: N/A Specificity: N/A Cohen’s Kappa: N/A AUC: N/A True skill statistic: N/A |

## Supplementary Table 5. Search terms for Web of Science Core Collection on 03/05/24.

| Number | Search Terms | Results |
| --- | --- | --- |
| #1 | (TI=("Species distribution model*" OR "distribution model*" OR "SDM$" OR "Ecological niche model*" OR "niche$model*" OR "Bioclimatic model*" OR "Climate envelope" OR "Habitat model*" OR "Habitat suitability model*" OR "Spatial model*" OR "Correlative model*")) OR AB=("Species distribution model*" OR "distribution model*" OR "SDM$" OR "Ecological niche model*" OR "niche$model*" OR "Bioclimatic model*" OR "Climate envelope" OR "Habitat model*" OR "Habitat suitability model*" OR "Spatial model*" OR "Correlative model*") | 52,249 |
| #2 | (TI=("sample size*" OR "sampling size*")) OR AB=("sample size*" OR "sampling size*") | 154,258 |
| #3 | (TI=("accurac*" OR "accurate" OR "performance" OR "reliab*" OR "robust" OR "stable")) OR AB=("accurac*" OR "accurate" OR "performance" OR "reliab*" OR "robust" OR "stable") | 10,004,360 |
| #4 | #1 AND #2 | 473 |
| #5 | #1 AND #2 AND #3 | 279 |
| #6 | #1 AND #2 AND #3 and Article (Document Types) | 253 |

**Supplementary Table 6. Search terms for Scopus on 03/05/24.**

| Number | Search Terms | Results |
| --- | --- | --- |
| #1 | TITLE-ABS("Species distribution model*" OR "distribution model*" OR "SDM" OR "SDMs" OR "Ecological niche model*" OR "niche model*" OR "niche-model*" OR "Bioclimatic model*" OR "Climate envelope" OR "Habitat model*" OR "Habitat suitability model*" OR "Spatial model*" OR "Correlative model*") | 63,014 |
| #2 | TITLE-ABS("sample size*" OR "sampling size*") | 189,889 |
| #3 | TITLE-ABS("accurac*" OR "accurate" OR "performance" OR "reliab*" OR "robust" OR "stable") | 13,997,331 |
| #4 | #1 AND #2 | 551 |
| #5 | #1 AND #2 AND #3 | 318 |
| #6 | #1 AND #2 AND #3 AND ( LIMIT-TO ( DOCTYPE , "ar" ) ) | 279 |

**Supplementary Table 7. Search terms for PubMed on 03/05/24.**

| Number | Search Terms | Results |
| --- | --- | --- |
| #1 | ((((((((((("Species distribution model*"[Title/Abstract]) OR ("distribution model*"[Title/Abstract])) OR (SDM[Title/Abstract])) OR ("Ecological niche model*"[Title/Abstract])) OR ("niche model*"[Title/Abstract])) OR ("niche-model*"[Title/Abstract])) OR ("Bioclimatic model*"[Title/Abstract])) OR ("Climate envelope"[Title/Abstract])) OR ("Habitat model*"[Title/Abstract])) OR ("Habitat suitability model*"[Title/Abstract])) OR ("Spatial model*"[Title/Abstract])) OR ("Correlative model*"[Title/Abstract]) | 14,906 |
| #2 | ("sample size*"[Title/Abstract]) OR ("sampling size*"[Title/Abstract]) | 109,098 |
| #3 | (((((accurac*[Title/Abstract]) OR (accurate[Title/Abstract])) OR (performance[Title/Abstract])) OR (reliab*[Title/Abstract])) OR (robust[Title/Abstract])) OR (stable[Title/Abstract]) | 3,519,744 |
| #4 | #1 AND #2 | 151 |
| #5 | #1 AND #2 AND #3 | 61 |

**Supplementary Table 8**. **Inclusion and exclusion criteria**

| **Criteria** | **Inclusion** | **Exclusion** |
| --- | --- | --- |
| **Population** | Terrestrial and simulated species and/or their pathogens (bacterial, parasitic, and viral) | Aquatic species or studies which do not predict species distribution (e.g., predicted soil content) |
| **Study design:** Scale | Large-scale modelling, defined as extents ≥ 100km x 100km and fine resolution ≤ 1km x 1km | Small-scale modelling, defined as extents < 100km x 100km and coarse resolution > 1km x 1km |
| **Study design:** Model & algorithm | Correlative species distribution models developed with Random Forest and presence-absence data  Models developed for a single species | Mechanistic species distribution models or correlative models developed with other algorithms (statistical or machine learning) using presence only, background points or pseudoabsences  Models developed for multiple species (e.g., joint species distribution models) or combined models for single species (e.g., ensemble and stacked models) |
| **Study design:** Covariates | Distribution described by environmental covariates | Distribution described by alternate covariates |
| **Outcome** | Studies which evaluate the sole effect of sample size on model performance as one of their aims | Studies which do not quantify the effect of sample size but describe general trends  Studies which evaluate multiple variables but do not separate the effect of sample size on model performance  Studies which predict species abundance |
| **Publication type** | Primary peer-reviewed publications | Grey literature or non-primary publications such as literature reviews, editorials, opinion pieces and position statements |
| **Geographic region & publication date** | Publications in any region until the search date (03/05/24) | No restrictions on geographic region or publication date |
| **Language** | Studies published in English | Studies published in a language other than English |

# Supplementary references

1. Page MJ, McKenzie JE, Bossuyt PM, Boutron I, Hoffmann TC, Mulrow CD, et al. The PRISMA 2020 statement: an updated guideline for reporting systematic reviews. *BMJ.* (2021) 372:n71. doi: 10.1136/bmj.n71.

2. RCoreTeam. R: A Language and Environment for Statistical Computing. (2023). Available online at: <https://www.R-project.org/> [Accessed: August 30, 2023].

3. Hijmans RJ. Terra: Spatial Data Analysis, R package version 1.7-39. (2023). Available online at: <https://CRAN.R-project.org/package=terra> [Accessed: August 30, 2023].

4. Liaw A, Wiener M. Classification and Regression by randomForest. *R News.* (2002) 2:18 - 22.

5. Freeman EA, Moisen G. PresenceAbsence: An R Package for Presence-Absence Analysis. *Journal of Statistical Software.* (2008) 23:1-31. doi: 10.18637/jss.v023.i11.

6. Kuhn M. Building Predictive Models in R Using the caret Package. *Journal of Statistical Software.* (2008) 28:1–26. doi: 10.18637/jss.v028.i05.

7. Meyer D, Zeileis A, Hornik K. vcd: Visualizing Categorical Data, R package version 1.4-11. (2023). Available online at: <https://CRAN.R-project.org/package=vcd> [Accessed: 30/08/2023].

8. Robin X, Turck N, Hainard A, Tiberti N, Lisacek F, Sanchez J-C, et al. pROC: an open-source package for R and S+ to analyze and compare ROC curves. *BMC Bioinformatics.* (2011) 12:77. doi: 10.1186/1471-2105-12-77.

9. Wickham H, François R, Henry L, Müller K, Vaughan D. dplyr: A Grammar of Data Manipulation, R package version 1.1.2. (2023). Available online at: <https://CRAN.R-project.org/package=dplyr> [Accessed: 30/08/2023].

10. Neuwirth E. RColorBrewer: ColorBrewer Palettes, R package version 1.1-3. (2022). Available online at: <https://CRAN.R-project.org/package=RColorBrewer> [Accessed: 30/08/2023].

11. Wickham H. ggplot2: Elegant Graphics for Data Analysis. (2016). Available online at: <https://ggplot2.tidyverse.org/> [Accessed: 30/08/2023].

12. Hendrickx A, Marsboom C, Rinaldi L, Vineer HR, Morgoglione ME, Sotiraki S, et al. Constraints of using historical data for modelling the spatial distribution of helminth parasites in ruminants. *Parasite.* (2021) 28:46. doi: 10.1051/parasite/2021042.

13. Liu Y, Zhang J, Ward MP, Tu W, Yu L, Shi J, et al. Impacts of sample ratio and size on the performance of random forest model to predict the potential distribution of snail habitats. *Geospatial Health.* (2023) 18:1151. doi: 10.4081/gh.2023.1151.

14. Liu C, Newell G, White M. The effect of sample size on the accuracy of species distribution models: considering both presences and pseudo-absences or background sites. *Ecography.* (2019) 42:535-48. doi: 10.1111/ecog.03188.

15. Grimmett L, Whitsed R, Horta A. Presence-only species distribution models are sensitive to sample prevalence: Evaluating models using spatial prediction stability and accuracy metrics. *Ecological Modelling.* (2020) 431:109194. doi: 10.1016/j.ecolmodel.2020.109194.

16. Hanberry BB, He HS, Dey DC. Sample sizes and model comparison metrics for species distribution models. *Ecological Modelling.* (2012) 227:29-33. doi: 10.1016/j.ecolmodel.2011.12.001.

17. Santini L, Benítez-López A, Maiorano L, Čengić M, Huijbregts MAJ. Assessing the reliability of species distribution projections in climate change research. *Diversity and Distributions.* (2021) 27:1035-50. doi: 10.1111/ddi.13252.

18. Shiroyama R, Wang M, Yoshimura C. Effect of sample size on habitat suitability estimation using random forests: a case of bluegill, *Lepomis macrochirus*. *Annales de Limnologie.* (2020) 56:13. doi: 10.1051/limn/2020010.

19. Soultan A, Safi K. The interplay of various sources of noise on reliability of species distribution models hinges on ecological specialisation. *PLoS ONE.* (2017) 12:e0187906. doi: 10.1371/journal.pone.0187906.

20. Tessarolo G, Rangel TF, Araújo MB, Hortal J. Uncertainty associated with survey design in Species Distribution Models. *Diversity and Distributions.* (2014) 20:1258-69. doi: 10.1111/ddi.12236.
