# Supplementary figures and images for: Predicting vector distribution in Europe: at what sample size are species distribution models reliable?
Source: Front Vet Sci. 2025 May 29;12:1584864. doi: 10.3389/fvets.2025.1584864 (PMC12159067; doi:10.3389/fvets.2025.1584864)

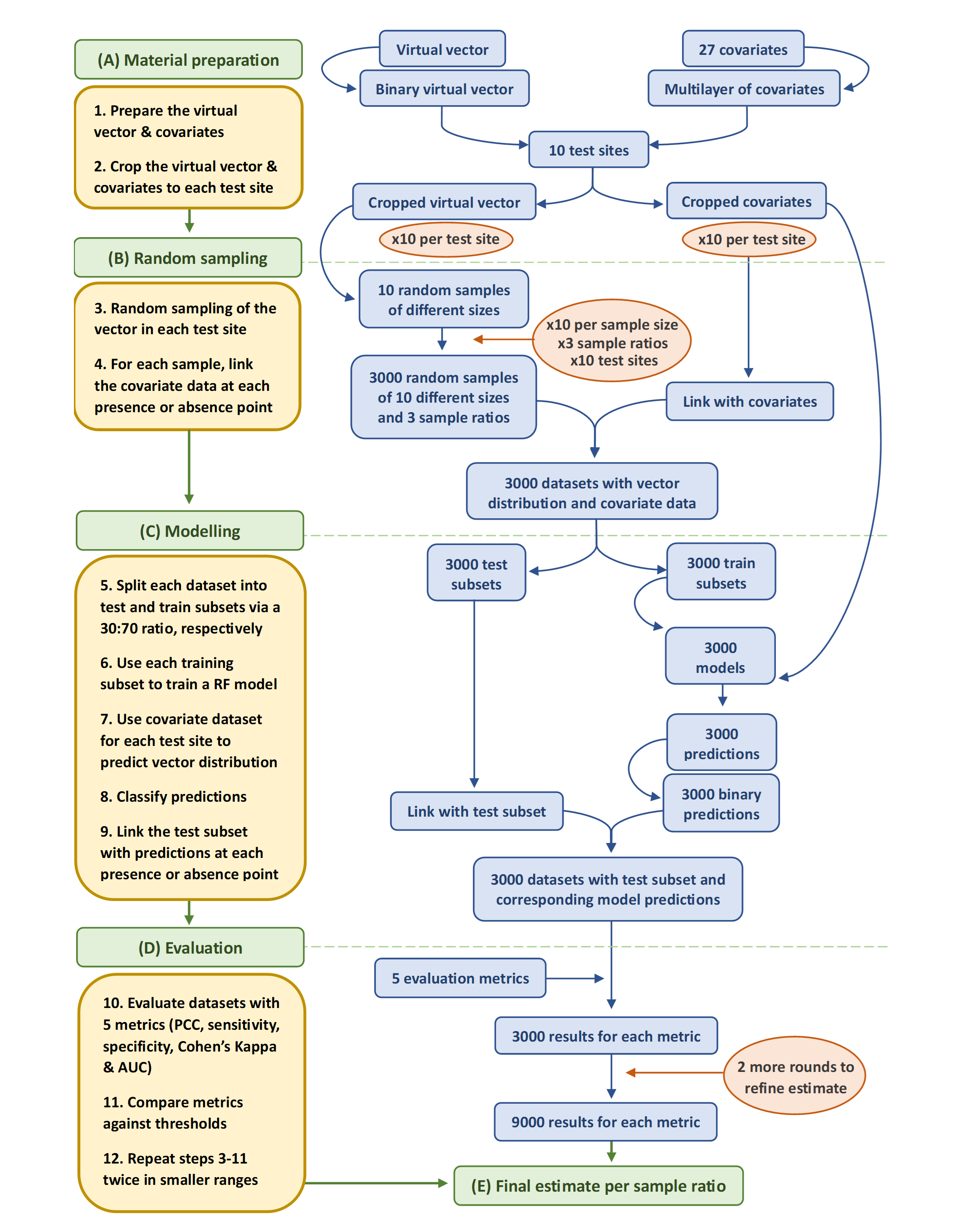

Supplement: Supplementary file 2 [file Presentation_1.zip › Supplementary Figure 1_Revised.tif]

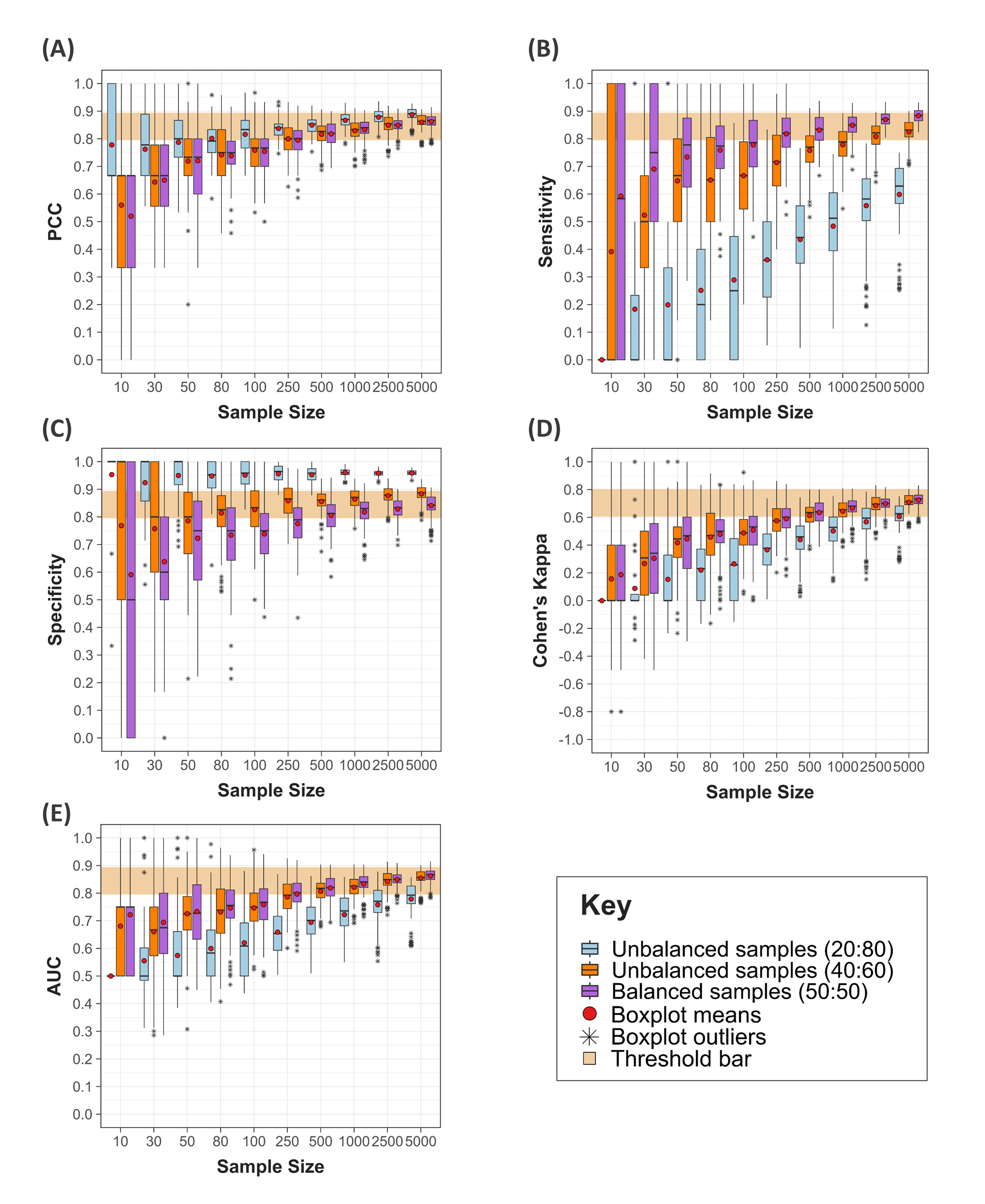

Supplement: Supplementary file 2 [file Presentation_1.zip › Supplementary Figure 2.tif]

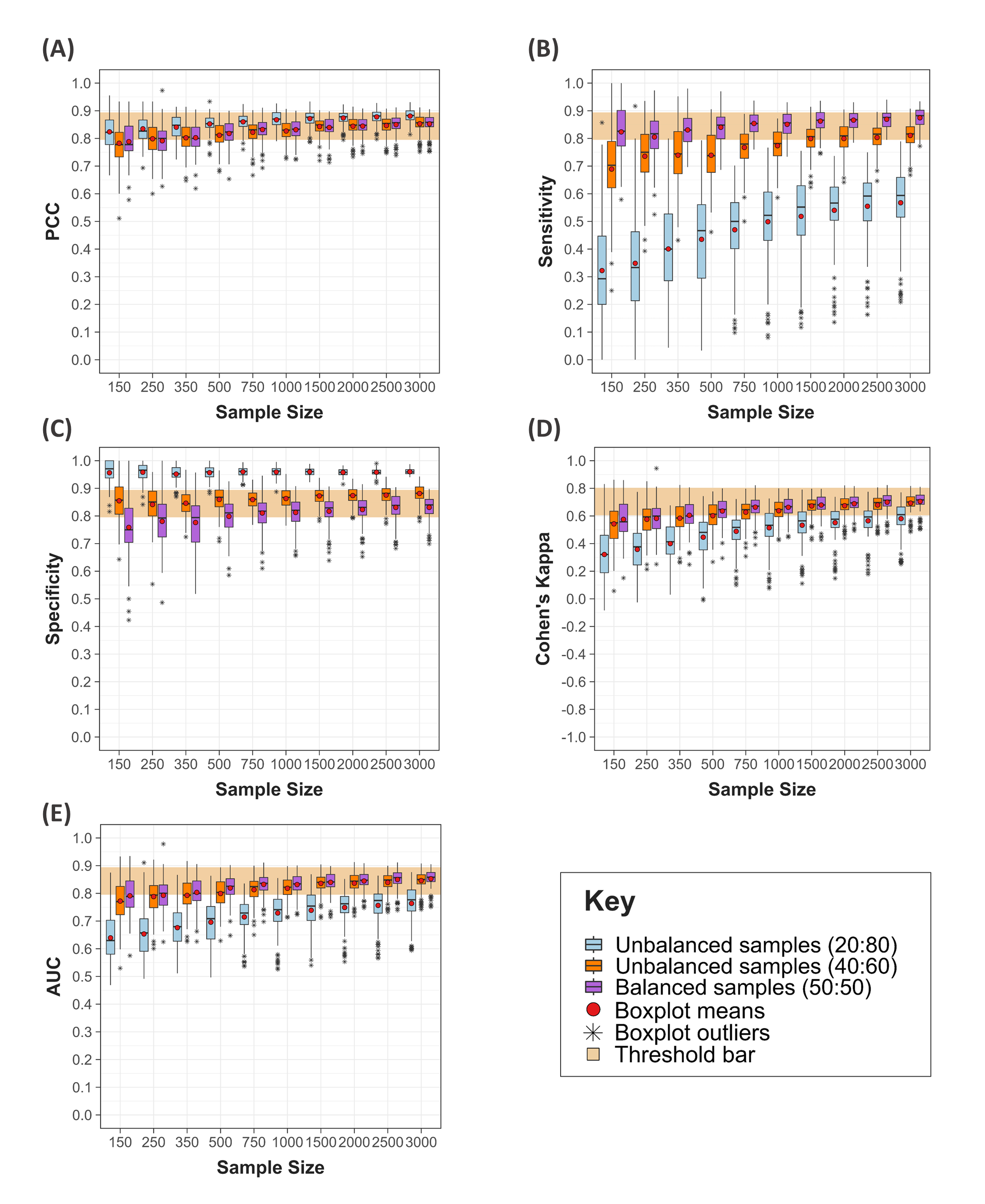

Supplement: Supplementary file 2 [file Presentation_1.zip › Supplementary Figure 3.tif]

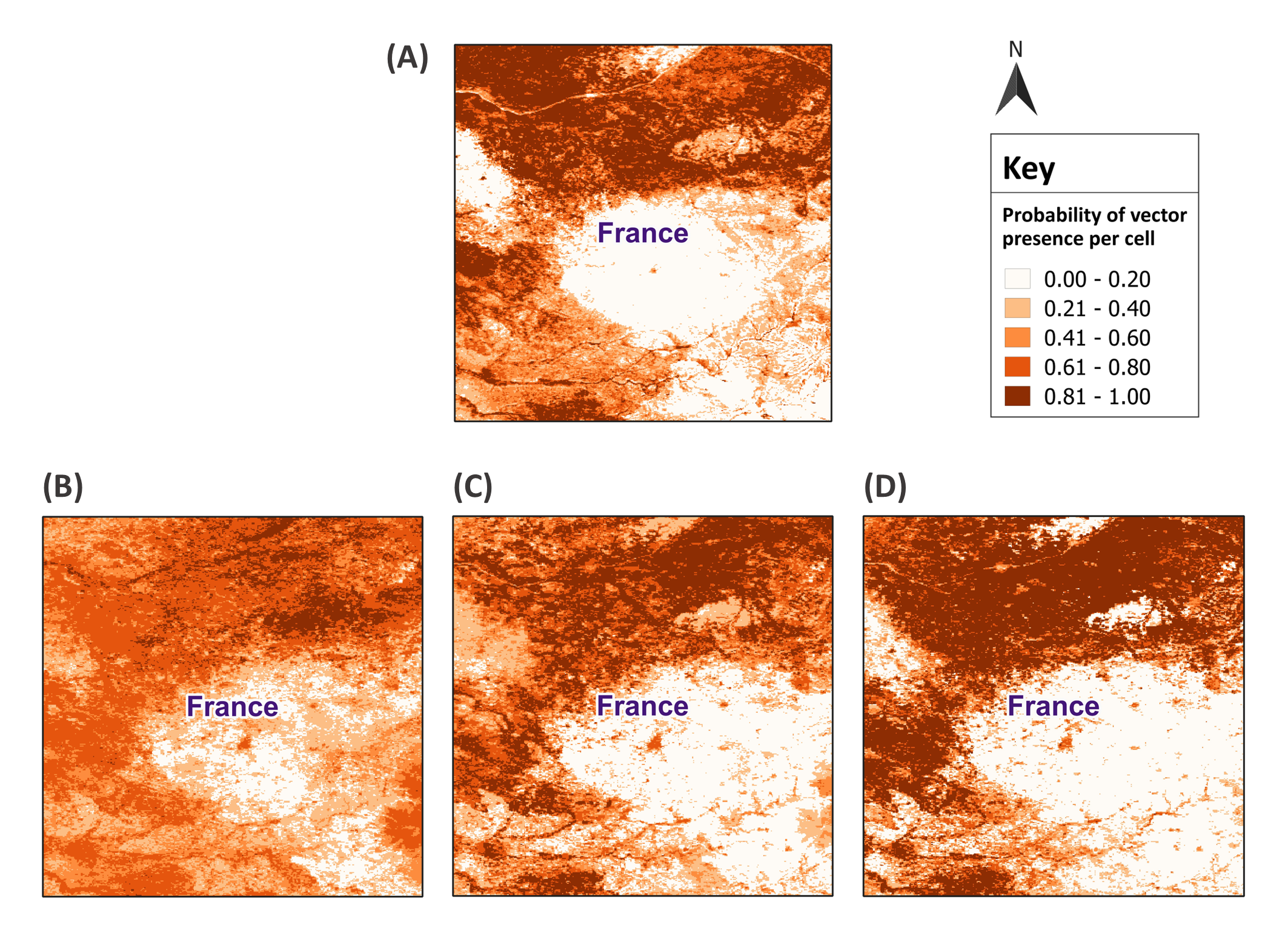

Supplement: Supplementary file 2 [file Presentation_1.zip › Supplementary Figure 4.tif]

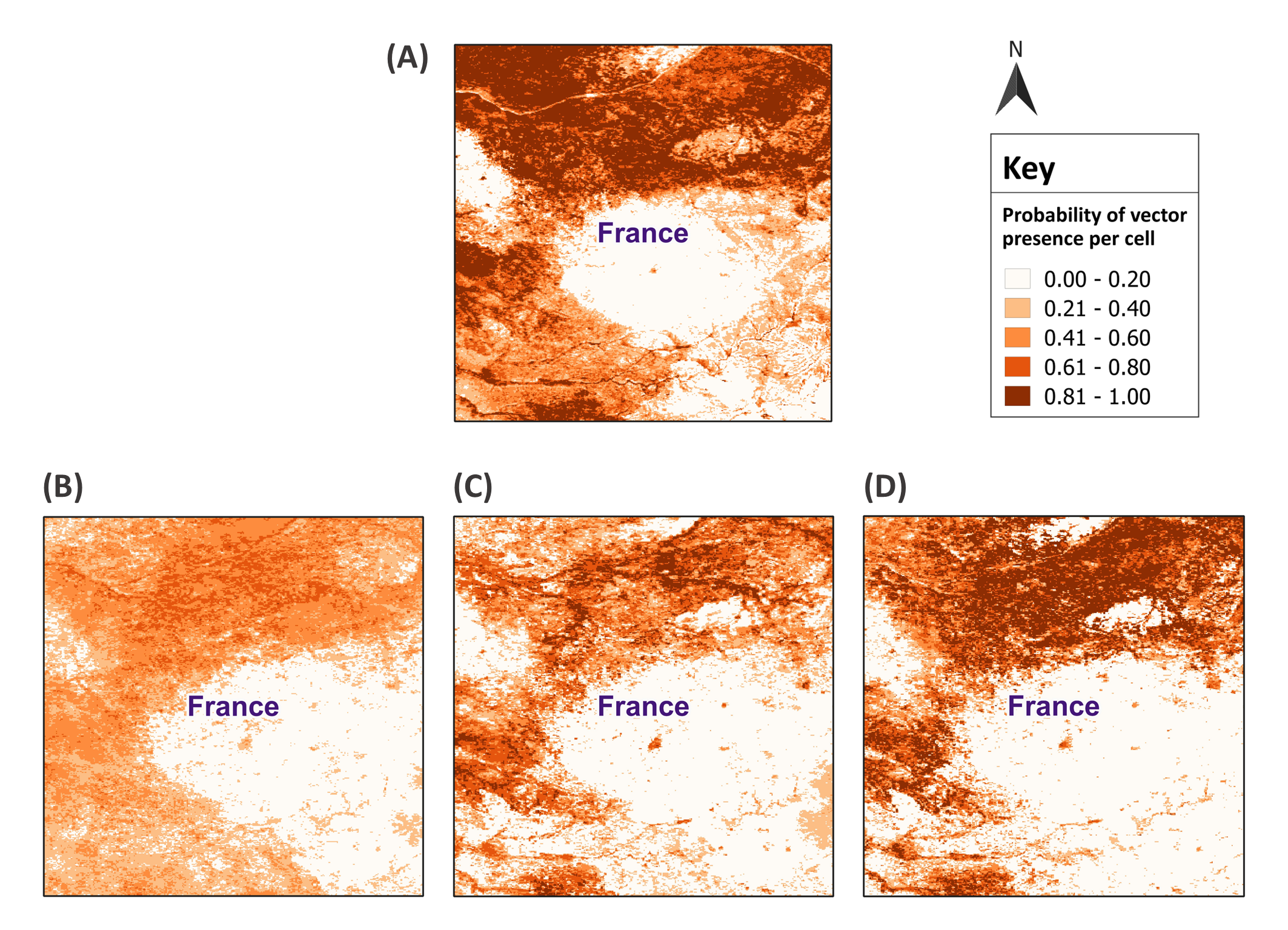

Supplement: Supplementary file 2 [file Presentation_1.zip › Supplementary Figure 5.tif]

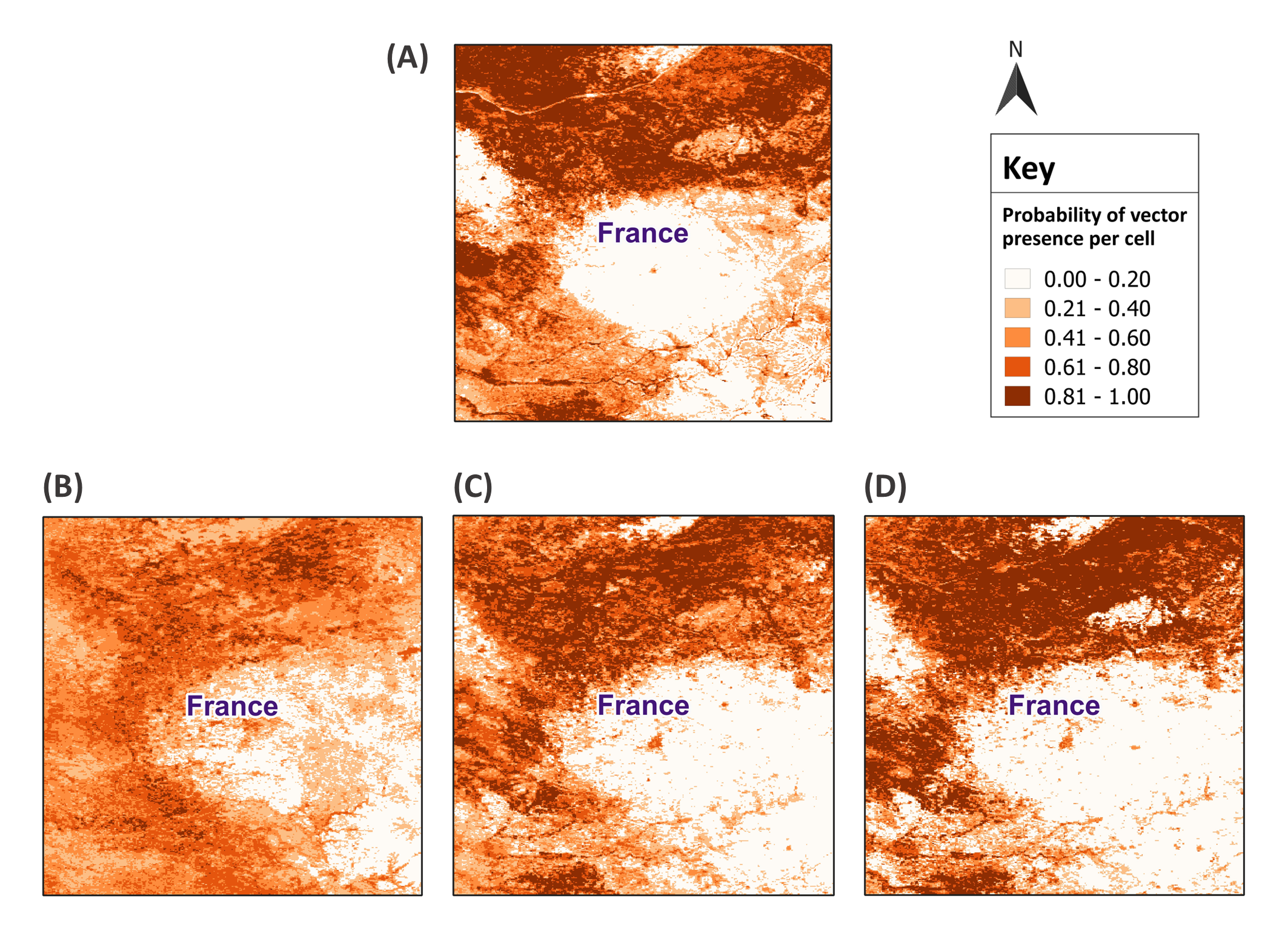

Supplement: Supplementary file 2 [file Presentation_1.zip › Supplementary Figure 6.tif]

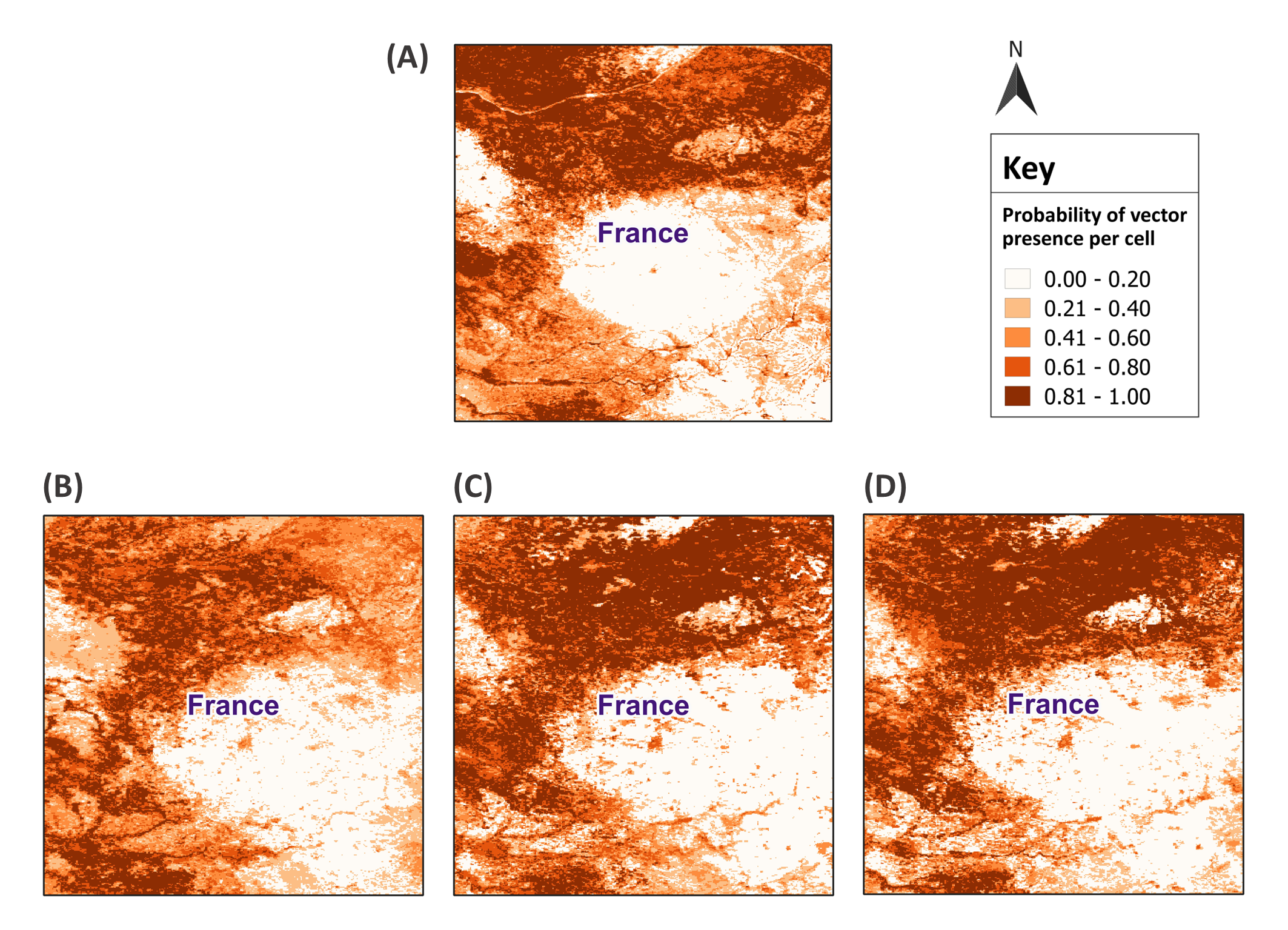

Supplement: Supplementary file 2 [file Presentation_1.zip › Supplementary Figure 7.tif]

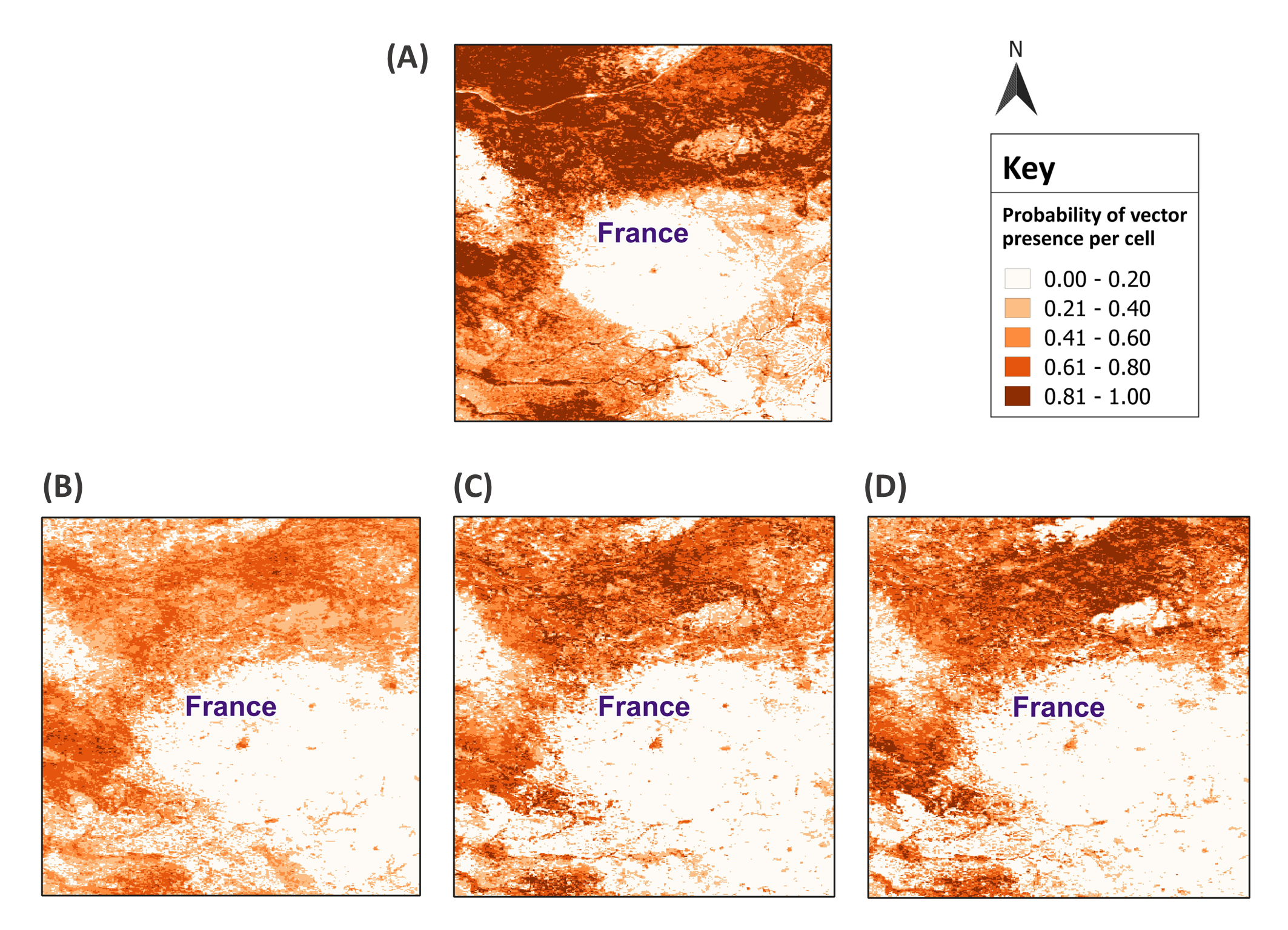

Supplement: Supplementary file 2 [file Presentation_1.zip › Supplementary Figure 8.tif]

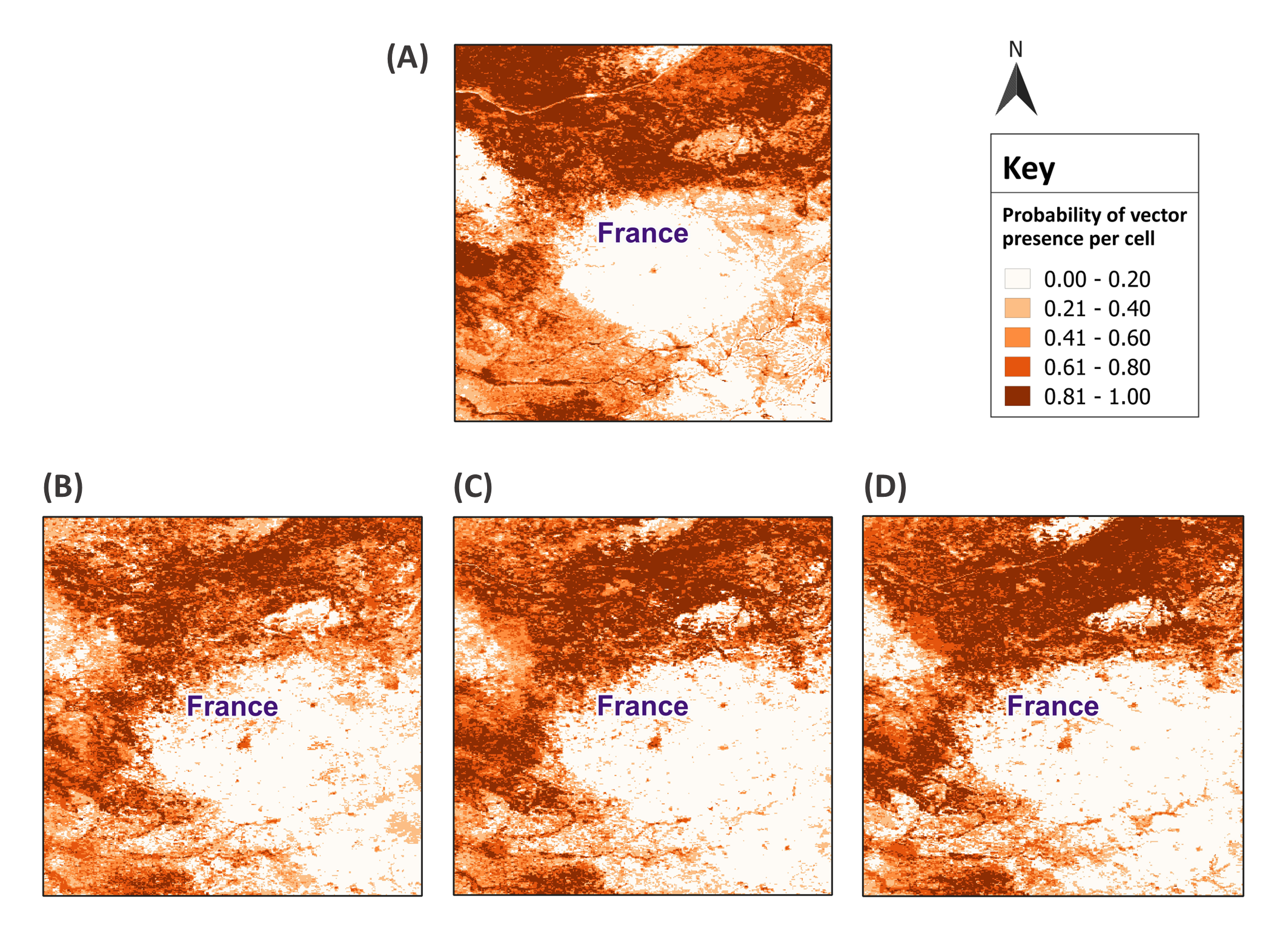

Supplement: Supplementary file 2 [file Presentation_1.zip › Supplementary Figure 9.tif]

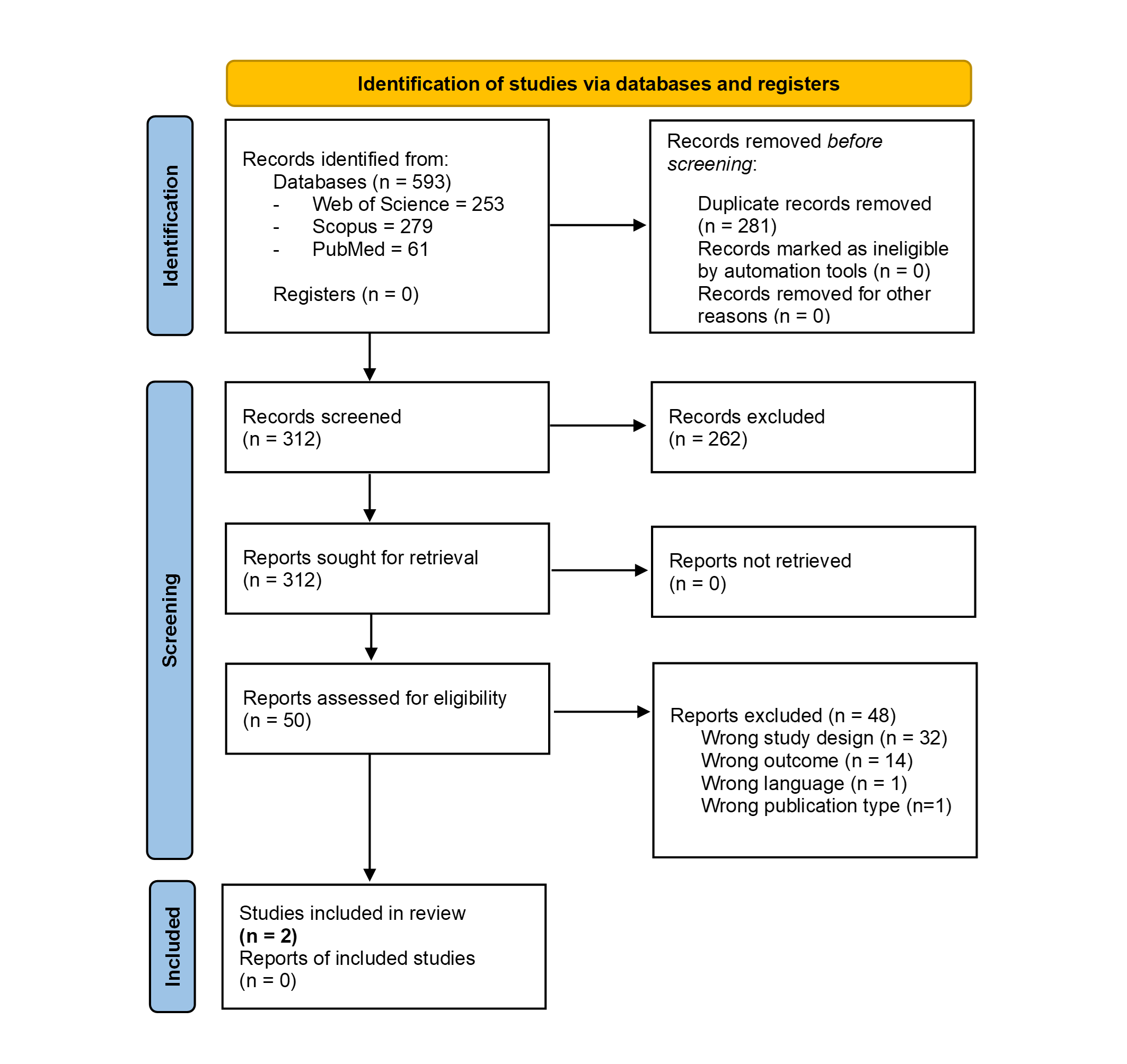

Supplement: Supplementary file 2 [file Presentation_1.zip › Supplementary Figure 10.tif]
